# *In Vivo* Electroporation Enhances the Immunogenicity of an HIV-1 DNA Vaccine Candidate in Healthy Volunteers

**DOI:** 10.1371/journal.pone.0019252

**Published:** 2011-05-16

**Authors:** Sandhya Vasan, Arlene Hurley, Sarah J. Schlesinger, Drew Hannaman, David F. Gardiner, Daniel P. Dugin, Mar Boente-Carrera, Roselle Vittorino, Marina Caskey, Johanne Andersen, Yaoxing Huang, Josephine H. Cox, Tony Tarragona-Fiol, Dilbinder K. Gill, Hannah Cheeseman, Lorna Clark, Len Dally, Carol Smith, Claudia Schmidt, Harriet H. Park, Jakub T. Kopycinski, Jill Gilmour, Patricia Fast, Robert Bernard, David D. Ho

**Affiliations:** 1 Aaron Diamond AIDS Research Center, New York, New York, United States of America; 2 The Rockefeller University, New York, New York, United States of America; 3 Ichor Medical Systems, Inc., San Diego, California, United States of America; 4 International AIDS Vaccine Initiative, New York, New York, United States of America; 5 International AIDS Vaccine Initiative, Human Immunology Laboratory, Imperial College, London, United Kingdom; 6 The EMMES Corporation, Rockville, Maryland, United States of America; University of Massachusetts Medical Center, United States of America

## Abstract

**Background:**

DNA-based vaccines have been safe but weakly immunogenic in humans to date.

**Methods and Findings:**

We sought to determine the safety, tolerability, and immunogenicity of ADVAX, a multigenic HIV-1 DNA vaccine candidate, injected intramuscularly by *in vivo* electroporation (EP) in a Phase-1, double-blind, randomized placebo-controlled trial in healthy volunteers. Eight volunteers each received 0.2 mg, 1 mg, or 4 mg ADVAX or saline placebo via EP, or 4 mg ADVAX via standard intramuscular injection at weeks 0 and 8. A third vaccination was administered to eleven volunteers at week 36. EP was safe, well-tolerated and considered acceptable for a prophylactic vaccine. EP delivery of ADVAX increased the magnitude of HIV-1-specific cell mediated immunity by up to 70-fold over IM injection, as measured by gamma interferon ELISpot. The number of antigens to which the response was detected improved with EP and increasing dosage. Intracellular cytokine staining analysis of ELISpot responders revealed both CD4+ and CD8+ T cell responses, with co-secretion of multiple cytokines.

**Conclusions:**

This is the first demonstration in healthy volunteers that EP is safe, tolerable, and effective in improving the magnitude, breadth and durability of cellular immune responses to a DNA vaccine candidate.

**Trial Registration:**

ClinicalTrials.gov NCT00545987

## Introduction

In 1993, Ulmer *et al.* first described the ability of naked plasmid DNA encoding an influenza protein to induce a protective immune response in mice [1], likely through transfection of myocytes and cross-presentation to antigen-presenting cells (APCs), as well as direct uptake of apoptotic cells by APCs [2]. Since then, DNA vaccines have been utilized in a variety of experimental clinical settings including candidate vaccines against cancer, malaria, hepatitis B, and HIV-1 [3]–[7]. Unfortunately, the robust cellular and humoral immunogenicity elicited by standard intramuscular injection of DNA vaccines in small animals has not translated to humans, as stand alone DNA vaccines have been weakly immunogenic in clinical trials. Although a few DNA vaccines have been licensed for use in animals, there are currently no DNA vaccines licensed for human use [8]. Consequently, the focus of many DNA vaccine strategies has shifted to their ability to “prime” the immune response before boosting with a recombinant live viral vector, such as adenovirus or modified vaccinia Ankara (MVA) [9]–[12], or with protein [13].

DNA vaccines offer several advantages over vaccines based on recombinant live viral vectors. Subjects may have pre-existing immunity to the viral vector itself, as in the case with adenovirus serotype 5-based vaccines, thereby limiting their effectiveness [14], [15]. Anti-vector immunity also develops rapidly after vaccination with recombinant viral vectors, effectively limiting the number administrations [16]. DNA vaccines are not limited by such constraints, and can be safely administered repeatedly to humans [17]. In addition, DNA vaccines can be rapidly produced using relatively simple, low-cost manufacturing procedures and exhibit a favorable thermostability profile. Such features would confer obvious advantages in large-scale global vaccination campaigns.

One major factor thought to contribute to the weak immunogenicity of DNA vaccines in humans is the relatively poor uptake of the vaccine by myocytes and other cells when injected intramuscularly (IM) [18]. *In vivo* electroporation (EP) is a technique that significantly increases the immunogenicity of DNA vaccines via co-administration of small, localized electrical fields to increase the transfection efficiency of the injected DNA [19], [20] and the recruitment of immune cells such as dendritic cells, T and B lymphocytes to the site of immunization [21], [22]. Animal studies in animals have shown that *in vivo* EP increases the immunogenicity of DNA vaccines encoding a number of antigens [23]–[32]. In humans, *in vivo* EP has been successful at delivering chemotherapeutic agents directly to tumors [33]. More recently, DNA vaccines encoding tumor antigens have been administered to cancer patients by EP as potential immunotherapy [34], [35].

ADVAX is a clade C/B' DNA vaccine candidate against HIV-1 [36]. When previously administered IM as a three vaccination regimen without EP to healthy volunteers at three different dosage levels, it proved to be safe but weakly immunogenic, inducing low-level, transient cellular responses, but no humoral response [37]. In this study, we sought to determine whether intramuscular administration of ADVAX via *in vivo* EP would be safe, tolerable and acceptable in healthy volunteers, and whether EP delivery would enhance immunogenicity compared to standard IM injection.

## Methods

### Study Setting

The study was conducted at the Rockefeller University Hospital in New York City, USA.

### Participants

Healthy men and women aged 18–60 years were eligible for participation if they were not at high risk for HIV-1, as defined by having none of the following activities in the six months prior to enrollment: unprotected vaginal or anal sex with a known HIV-1-infected person or casual partner, injection drug use, acquisition of a sexually transmitted disease, or sex work for money or drugs. Participants agreed to safe sexual practices and to effective contraception to avoid pregnancy throughout the duration of the 14-month study. Participants had to demonstrate a clear understanding of the possibility of HIV-1 seropositivity due to vaccine-induced antibodies. Exclusion criteria included chronic medical conditions, clinically significant abnormal laboratory parameters, infection with Hepatitis B or C virus, recent receipt of a vaccine or blood transfusion, any implanted electronic stimulation device, or deltoid skin fold thickness of greater than 40 mm.

### Ethics Statement

The study was approved by the Institutional Review Board of the Rockefeller University Hospital. All participants in this study provided written informed consent after appropriate review, discussion and counseling by the clinical study team. The trial was conducted in partnership with Ichor Medical Systems, Inc. and the International AIDS Vaccine Initiative (IAVI), and sponsored by the Bill and Melinda Gates Foundation Collaboration for AIDS Vaccine Discovery. The study was conducted in compliance with International Conference on Harmonisation - Good Clinical Practice (ICH-GCP) guidelines.

### Interventions

#### Candidate Vaccine

The ADVAX vaccine candidate is a 1∶1 mixture of two DNA plasmids containing clade C/B', codon-optimized HIV-1 gene sequences. The first plasmid expresses Env under the CMV promoter and Gag under the human elongation factor 1α (PhEF1α) promoter, while the second expresses Pol under the CMV promoter and a Nef-Tat fusion under the PhEF1α promoter, as previously described [36]. A Phase-1 clinical trial of ADVAX injected IM has been reported previously [37].

#### Electroporation Procedure

The disposable electroporation cartridge was loaded with placebo or ADVAX by the Rockefeller University Hospital Pharmacy and then adjusted to one of three depth settings, corresponding to pre-defined ranges in skin fold thickness. The cartridge was loaded into the EP device and applied to the deltoid muscle. Intramuscular administration of ADVAX or placebo was followed immediately by the application of electrical stimulation (TriGrid^TM^ Delivery System, Ichor Medical Systems, San Diego, CA). The spacing of the TriGrid^TM^ electrode array was 6 mm in a diamond-shaped configuration, and the electrical field was applied at an amplitude of 250 V/cm of electrode spacing for a 40 msec total duration applied as three pulses over a 400 msec interval, resulting in brief deltoid muscle contractions. All electroporation procedures were performed by a single, trained physician.

#### Study Design

The study design is summarized in Table 1. This study was randomized, dose-escalating, and double blind with respect to active vaccine candidate or saline placebo, but not with respect to dose group or mode of administration (IM versus EP). The randomization schedule was prepared by the Rockefeller University Hospital Pharmacy, using a web-based program at randomization.com. Each of the 3 cohorts consisted of 2 or 3 subjects randomized to receive 4 mg ADVAX IM (HD-IM), 2 or 3 subjects randomized to receive placebo EP, and 8 subjects receiving ADVAX EP. The dose of ADVAX delivered by EP varied with each cohort in a dose-escalating design: 0.2 mg (LD-EP), 1 mg (MD-EP) or 4 mg (HD-EP). Dosage levels were based on previously tested concentrations of ADVAX [37], with the intent to determine whether EP delivery of ADVAX provided any dose-sparing effect as measured by immunogenicity. In total, 40 subjects were enrolled. Blinded safety and tolerability in each cohort were evaluated by an independent Data and Safety Monitoring Board in a blinded manner prior to initiation of enrollment of the next dosage cohort. After all volunteers had received both scheduled vaccinations, the trial was amended to include a third vaccination at week 36 in volunteers randomized to receive EP in the high-dose group cohort (n = 8 ADVAX, n = 3 placebo), in order to determine whether a third vaccination at the highest dose could further enhance immunogenicity. The protocol for this trial and supporting CONSORT checklist are available as supporting information; see Checklist S1 and Protocol S1.

**Table 1:** Study Design.

	Placebo (Saline/EP)	ADVAX IM (4.0 mg)	ADVAX EP
**Group 1**	2	2	8 (0.2 mg)
**Group 2**	3	3	8 (1.0 mg)
**Group 3**	3	3	8 (4.0 mg)
**Study Total**	**8**	**8**	**24**

### Objectives

The primary objective was to evaluate the safety and tolerability of ADVAX delivered intramuscularly via *in vivo* EP at one of three dose levels versus ADVAX delivered by standard intramuscular injection and placebo delivered via EP in healthy HIV-uninfected adults. The secondary objective was to evaluate the humoral and cellular immunogenicity of ADVAX-EP versus ADVAX-IM.

### Outcomes

#### Vaccine Reactogenicity, Safety, Tolerability, and Acceptability

Primary endpoints were designed to evaluate the safety of ADVAX in human volunteers. Local reactogenicity (including pain, tenderness, erythema, edema, skin damage, induration, and formation of crust, scab or scar) and systemic reactogenicity (including fever, chills, headache, nausea, vomiting, malaise, myalgia, arthralgia, and rash) were assessed within 30–45 minutes after each vaccination in the clinic, by telephone three days following vaccination, and by history and physical examination one week after vaccination. Subjects were monitored for adverse events, general health and clinical laboratory parameters at each study visit. Subjects randomized to receive ADVAX or placebo via EP were asked to complete a questionnaire rating their pain on a five-point scale at three time points during and after the EP procedure, as well as the perceived acceptability of the procedure for use in the setting of preventive immunization 30–45 minutes after each vaccination.

#### Immunological Analyses

Secondary endpoints evaluated the cellular and humoral immunogenicity of ADVAX at 0, 1, 2, and 4 weeks after each vaccination as well as at weeks 16, 24, 36, 48, and 56. Cellular immunogenicity was assessed by IFNγ ELISpot on frozen peripheral blood mononuclear cells (PBMCs) stimulated by peptides matched to the Clade C/B' sequences encoded in the vaccine as previously described [37].

For each pool, the ELISpot value was defined as the mean replicate (maximum 4) count minus the mean background count. Four criteria had to be fulfilled for an ELISpot value to be considered positive: 1) for each peptide pool, a single value had to be greater than the maximum of all pre-vaccination and all placebo values for that pool, and >38 Spot Forming Units (SFU)/10^6^ cells; 2) the mean count had to be >4 times the mean background SFU; 3) the mean background had to be <55 SFU/10^6^ cells; and 4) the coefficient of variation had to be ≤70% across the replicate wells.

ELISpot-positive samples at the peak responding time point in the high-dose EP group, along with the respective baseline samples, were tested for phenotype, cytokine secretion, and antigen-specific proliferation using polychromatic flow cytometry. Cryopreserved PBMCs were co-incubated with 2 µg peptide pools or 1 µg SEB (Sigma-Aldrich, St. Louis, MO), CD107 PECy5 (Becton Dickinson, San Jose, CA), Brefeldin A (Sigma-Aldrich, Poole Dorset, UK) and BD Golgistop (Becton Dickinson, San Jose, CA) for 6 hours at 37°C. Cells were stained with 50 µL LIVE/DEAD® Fixable Blue Dead Cell Stain Kit (Invitrogen, Eugene, OR), anti-CD4 QD605, anti-CD8 pacific orange (Invitrogen, Paisley, UK), anti-CD27 FITC (Becton Dickinson, San Jose, CA), and anti-CD45RO (Beckman Coulter, High Wycombe, UK), and stained intracellularly with anti-CD3 QD655 (Invitrogen, Paisley, UK), anti-IFN-γ PE Cy7, anti-MIP-1β PE, anti-TNF-α A700 and anti-IL-2 APC (Becton Dickinson, San Jose, CA). At least 500,000 events were acquired on a custom-built BD LSR II cytometer. Data were analyzed using FlowJo (Treestar), PESTLE and SPICE (courtesy of Mario Roederer, Vaccine Research Center) software. A response was considered positive if it fulfilled the following three criteria: 1) the percentage of cytokine-producing cells after antigen stimulation was at least three times greater than the percentage of cytokine-producing cells in the mock pool at the same post-vaccination time point, 2) the response to the same antigen was negative at pre-vaccination baseline, and 3) the absolute response was ≥0.05%.

#### Humoral immunogenicity

Binding antibodies to clade C gp120 (NIH AIDS Reagent Program) were assessed by ELISA at pre-vaccination baseline and two weeks after each vaccination, as previously described [37]. In parallel, anti-gp160, anti-p24, or anti-gp36 Group M/O antibodies were assessed using the Genetic Systems^TM^ HIV-1| HIV-2 PLUS O EIA Kit (Bio-Rad Laboratories, Hercules, CA), at the New York State Department of Health. Samples that were positive were further evaluated by the Genetic Systems^TM^ HIV-1 Western Blot Kit (Bio-Rad Laboratories, Hercules, CA) and for viral load quantification using the Roche Amplicor HIV-1 Monitor v1.5 RNA-PCR Kit (Roche Diagnostic Systems, Indianapolis, IN) to differentiate a response to vaccine from incident HIV-1 infection. Results were monitored by an independent physician to maintain blinding of the clinical study team.

### Statistical Methods

**Figure 1:**
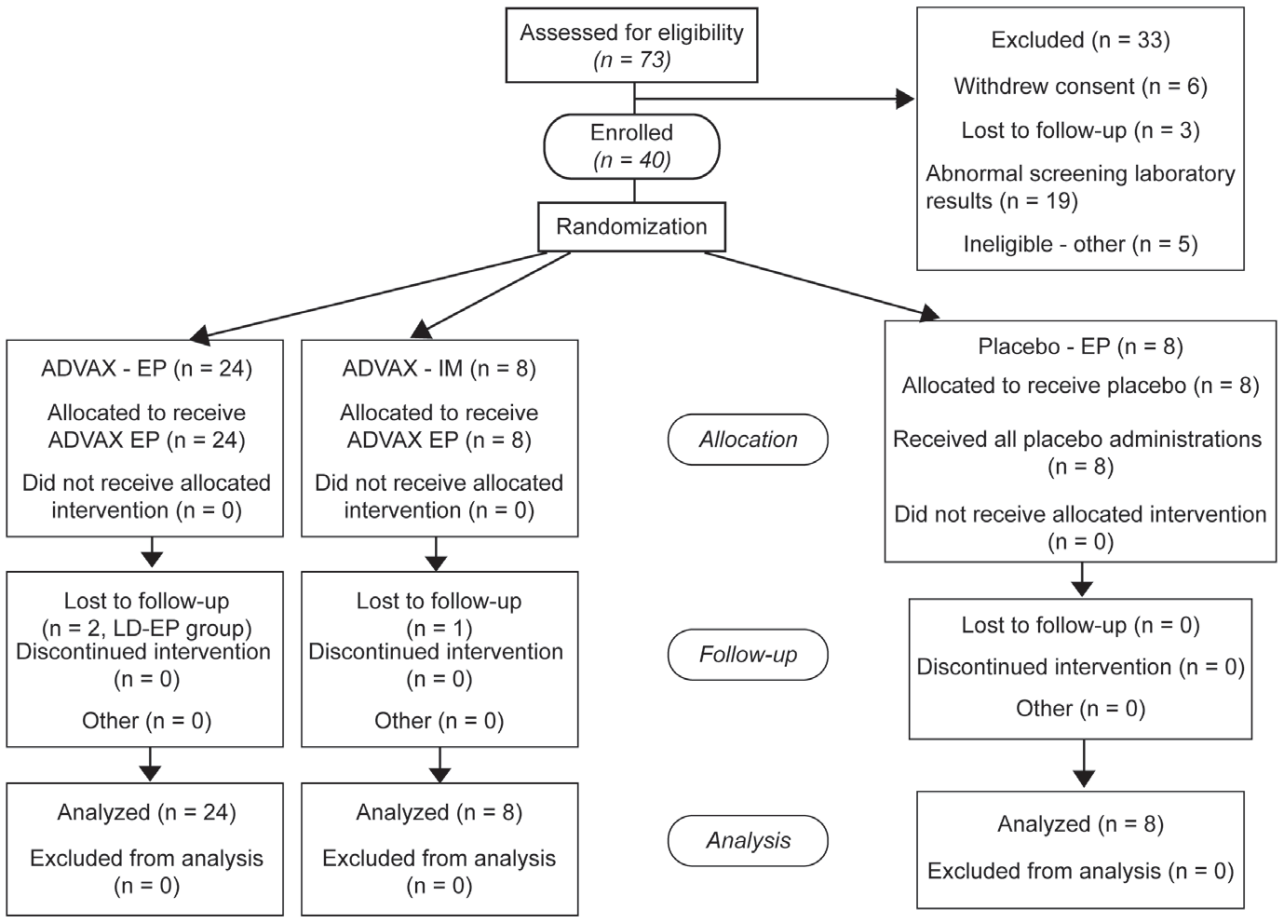
Participant Flow Diagram.

Data from all participants, including those lost to follow up and those not completing the vaccination series, were included in the analyses, as per the participant flow diagram in Figure 1. Fisher's exact test was used to test differences in the rate of local and systemic reactogenicity events between groups, and the Cochran-Armitage test was used to investigate trends in event rates with increasing ADVAX EP dosage. Differences in magnitude of ELISpot responses between each EP dose group and the IM group were analyzed using the non-parametric Wilcoxon 2-sample test (t approximation), with significance set at p<0.017 to allow for three tests per antigen. All tests are 2-tailed.

## Results

### Recruitment and Participant Flow

Enrollment occurred from October 2007 through October 2008. As shown in Figure 1, 73 volunteers were screened for this study, of which 40 were enrolled. The majority of the 33 screen failures were due to abnormalities on screening laboratories or urinalysis. All volunteers completed their vaccination schedule, but three participants did not complete the trial for reasons unrelated to the vaccine or the study (lost to follow-up). Baseline demographic and clinical characteristics for all trial participants are summarized in Table 2.

**Table 2:** Volunteer Demographics.

Method of Administration		Electroporation				IM	Total
**ADVAX Dose (mg)**		**0.2**	**1.0**	**4.0**	**Placebo**	**4.0**	
**Number of Volunteers**		8	8	8	8	8	40
**Timing of Administration (week)**		0, 8	0, 8	0, 8, 36*	0, 8, 36*	0, 8	N/A
**Gender**	Male	6	3	3	2	7	21
	Female	2	5	5	6	1	19
**Ethnicity**	**Race**						
**Not Hispanic and Not Latino**	White	5	6	3	5	3	22
	Black or African American	1	0	5	2	1	9
	Multiracial	0	1	0	0	1	2
	**Total**	6	7	8	7	5	33
**Hispanic or Latino**	**Race**						
	White	1	1	0	0	2	4
	Multiracial	1	0	0	0	0	1
	Other/Unknown	0	0	0	1	1	2
	**Total**	2	1	0	1	3	7
**Age at enrollment (years)**	**Mean**	33.0	29.9	37.1	33.6	39.1	34.6
	**Range**	18–59	21–53	24–52	19–52	21–58	18–59
**Body Weight (kg)**	**Mean**	**76.8**	**68.4**	**78.4**	**82.6**	**77.0**	**76.6**
	**Range**	**58–100**	**52–85**	**46–115**	**53–120**	**50–99**	**46–120**

*Only those volunteers in the high dose cohort (HD-EP, n = 8, and Placbeo-EP, n = 3) received a 3^rd^ vaccination at Week 36.

### Reactogenicity and Adverse Events

Overall, ADVAX delivered by standard IM injection or by EP was safe and well-tolerated, although most volunteers in all dose groups reported mild-moderate local pain and/or tenderness. The proportion of volunteers with mild-moderate local pain and/or tenderness as assessed by the clinical study team within 30–45 minutes of vaccination differed significantly (p<.001) among the 5 dose groups, being smaller in the HD-IM group (2/8) than in any of the EP groups (6/8 EP-placebo and 8/8 each ADVAX EP group). There was no significant difference in self-assessed local reactogenicity within 4 days following the vaccination (p = 0.291). Most local reactions resolved within one day; all resolved within 7 days. The maximum severity of systemic reactogenicity events after any ADVAX or placebo administration was mild when assessed within 30–45 minutes of vaccination in clinic and moderate within 4 days following vaccination when assessed by the volunteer. All systemic reactions resolved within 2 days. Differences in systemic reactogenicity among the 5 study groups were not statistically significant (clinic: p = 0.252, self-assessment: p = 0.291).

Of the 139 non-serious adverse events, 123 (89%) were mild. One volunteer, who was in the MD-EP group, and who received saline placebo, experienced a serious adverse event (hospitalization for coronary artery disease) 109 days after his second vaccination, which was unrelated to study vaccine or procedure. None of the moderate or severe adverse events were related to vaccination, and none of the volunteers discontinued the study due to adverse events. The distribution of mild and moderate adverse events was not significantly different among the 5 dose groups (p = 0.414, Fisher's exact 2-tailed test). Table S1 summarizes all adverse events by System Organ Class (SOC). There were no differences in clinical laboratory parameters among study groups or trends within any study group over time. None of the volunteers developed anti-double-stranded DNA antibodies.

### Tolerability and Acceptability of Electroporation

**Figure 2:**
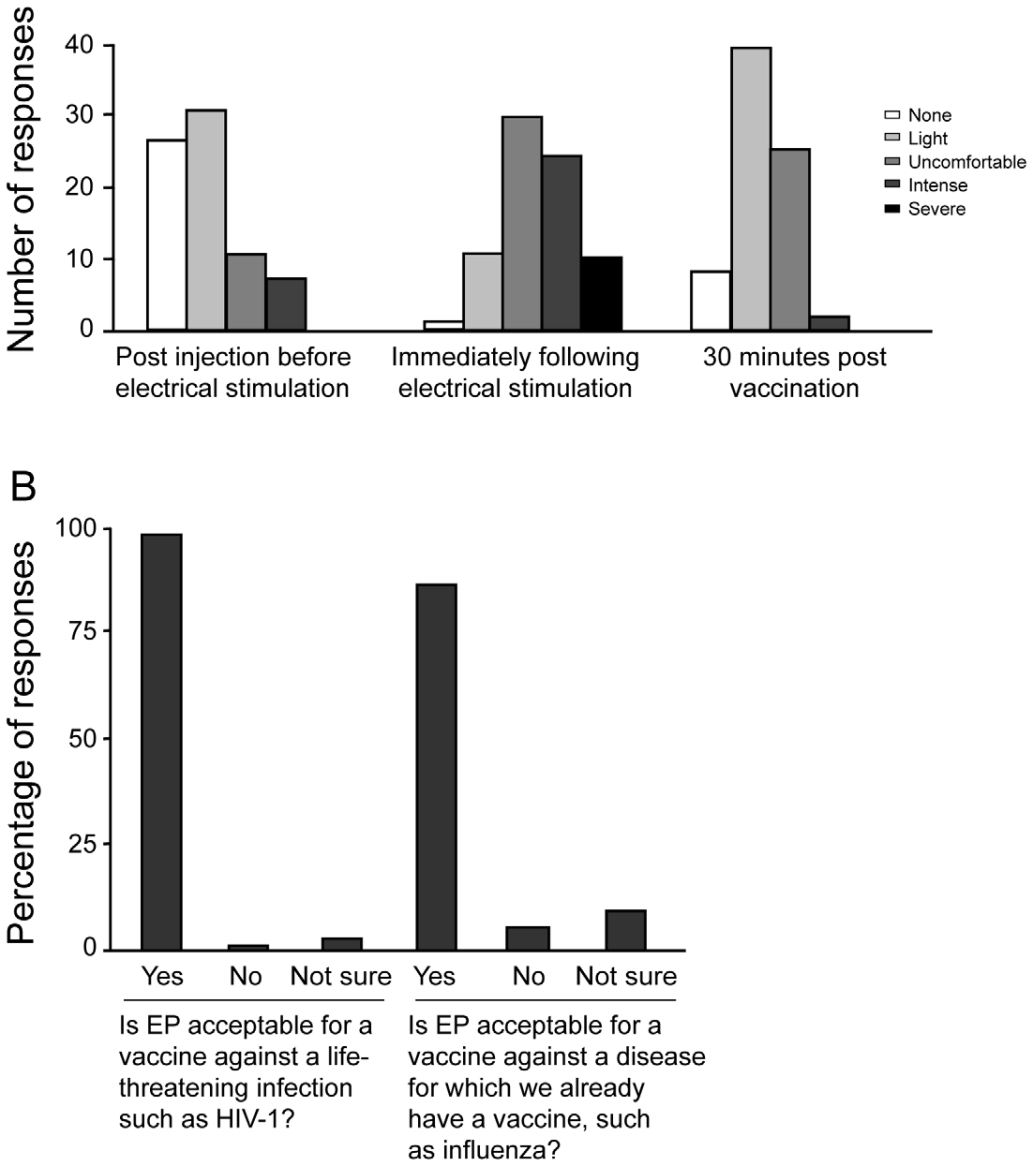
Tolerability and Acceptability of Electroporation. Volunteers randomized to receive ADVAX or placebo via EP completed a questionnaire to rate the tolerability of the procedure on a 5 point pain scale at three different time points during and after EP (Panel A), and the acceptability of the procedure for future vaccination (Panel B). Results represent a total of 75 responses from 32 volunteers.

Figure 2 summarizes the tolerability (Panel A) and acceptability (Panel B) of the electroporation procedure. The intensity of pain was greatest immediately after electrical stimulation of the muscle, but improved rapidly within 30 minutes post vaccination. For all 3 assessments, the proportions of volunteers reporting uncomfortable, intense or severe discomfort were not significantly different among the 4 EP dose groups. The level of tolerability was independent of age, gender, body weight, skin fold thickness, vaccination in dominant versus non-dominant arm, or sequence of vaccination. The majority of participants indicated that they would undergo the procedure for a vaccination to protect against either a life threatening illness for which we have no alternative vaccine such as HIV-1 (97%), or, to improve the protection achievable with existing vaccines against a non-life threatening illness such as influenza (91%).

### Cellular Immunogenicity

IFNγ ELISpot results are summarized in Table 3. Positive IFNγ ELISpot responses after two vaccinations occurred in 0/8 (0%), 1/8 (13%), 5/8 (63%), and 6/8 (75%) volunteers in the HD-IM, LD-EP, MD-EP and HD-EP groups, respectively. The response rate in the HD-EP group increased to 7/8 (88%) after the third vaccination. There were no positive responses to any peptide pool among the placebo recipients, by definition. There was a dose-dependent increase in the number of antigens to which a response was detected.

**Table 3:** Summary of IFNγ ELISpot Positive Responses.

Group		EP Placebo	IM High	EP Low	EP Mid	EP High
**ADVAX Dose**		0 mg	4.0 mg	0.2 mg	1.0 mg	4.0 mg
**Volunteers with Positive Responses**		0/8 (0%)	0/8 (0%)	1/8 (13%)	5/8 (63%)	7/8 (88%)
**Env** (SFU/million)	n	0	0	1	5	6
	mean	n/a	n/a	193	224	273
	median	n/a	n/a	193	201	275
	25–75%ile	n/a	n/a	n/a	176–229	186–336
	range	n/a	n/a	193	161–440	150–595
**Pol**(SFU/million)	n	0	0	0	2	5
	mean	n/a	n/a	n/a	56	84
	median	n/a	n/a	n/a	56	78
	25–75%ile	n/a	n/a	n/a	46–66	59–115
	range	n/a	n/a	n/a	39–74	44–158
**Gag**(SFU/million)	n	0	0	0	0	2
	mean	n/a	n/a	n/a	n/a	85
	median	n/a	n/a	n/a	n/a	83
	25–75%ile	n/a	n/a	n/a	n/a	68–95
	range	n/a	n/a	n/a	n/a	48–133
**Nef Tat**(SFU/million)	n	0	0	0	1	3
	mean	n/a	n/a	n/a	80	98
	median	n/a	n/a	n/a	80	96
	25–75%ile	n/a	n/a	n/a	n/a	82–115
	range	n/a	n/a	n/a	80	75–128

**Figure 3:**
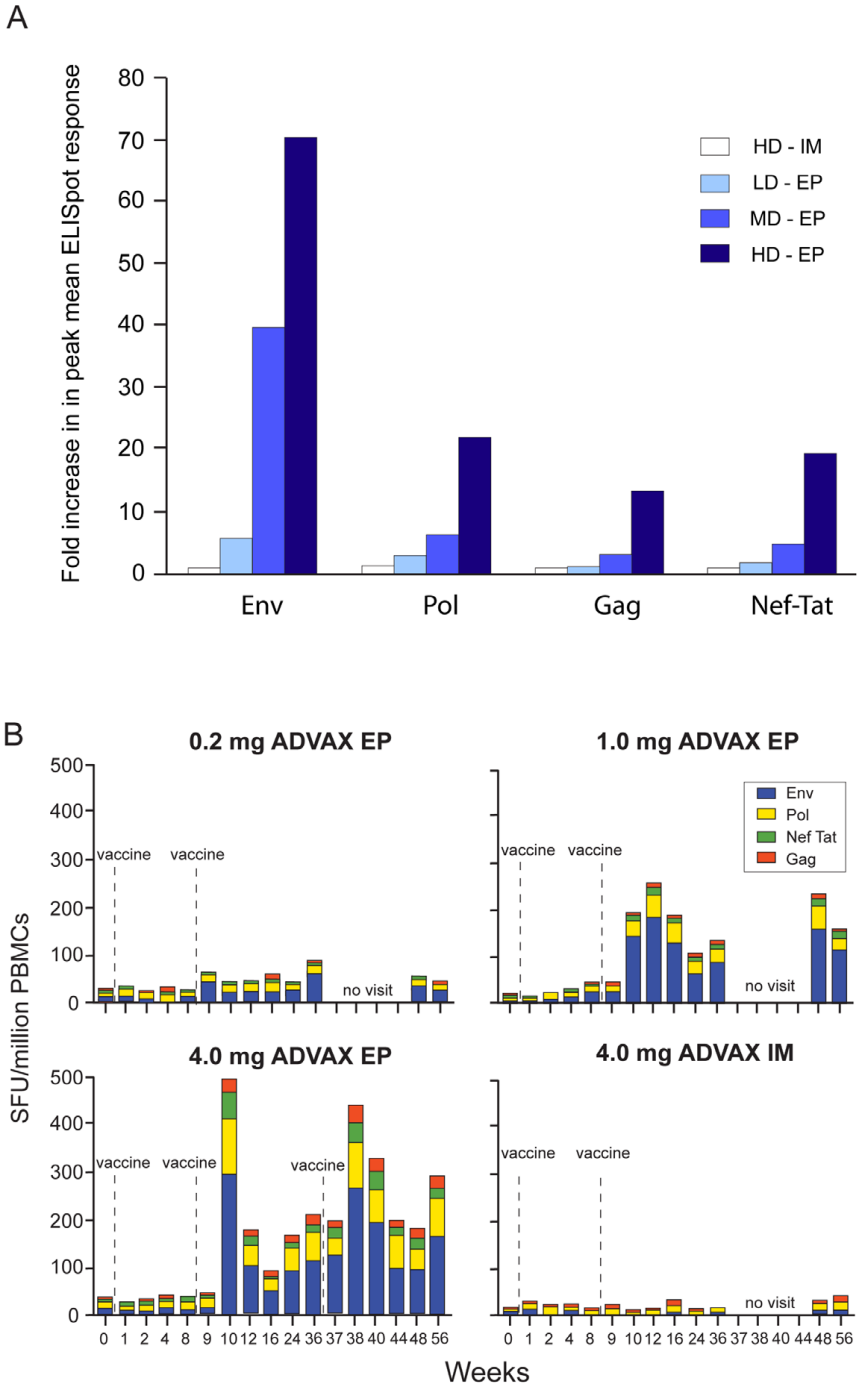
Cellular Immune Response. Panel A depicts the fold increase over the HD-IM response in the mean of all IFNγ ELISpot responses to each antigen at Week 10, coinciding with the peak cellular immune response. Panel B depicts the sum of all mean ELISpot counts for each peptide pool at each study time point for all ADVAX dose groups, color coded by antigen. SFU =  spot forming units.

As shown in Figure 3A, delivery of the same dose of ADVAX via EP (HD-EP) resulted in a 70-fold increase in the mean IFNγ ELISpot response to Env over the HD-IM response at Week 10, the time of peak cellular immune response after the second vaccination. Responses to the Pol, Gag, and Nef-Tat antigens in the HD-EP group increased by 22, 13, and 19 fold over the mean HD-IM IFNγ ELISpot, respectively. There was a clear dose response in the fold increase to each antigen, as the MD-EP IFNγ ELISPOT responses to Env, Pol, Gag, and Nef-Tat increased by 40, 7, 3, and 5-fold over HD-IM responses, respectively. There was no correlation between age of volunteer and magnitude of IFNγ ELISpot response within any of the groups.

Figure 3B depicts the sum of all mean IFNγ ELISpot background-subtracted counts for each antigen over time by dose group. The magnitude of response increased in the electroporation groups in a dose-dependent manner. The strongest IFNγ ELISPOT responses were to Env, and the weakest were to Gag. Responses persisted in 1/8 volunteers in the MD-EP and 2/8 volunteers in the HD-EP group until the end of the trial (Week 56).

**Figure 4:**
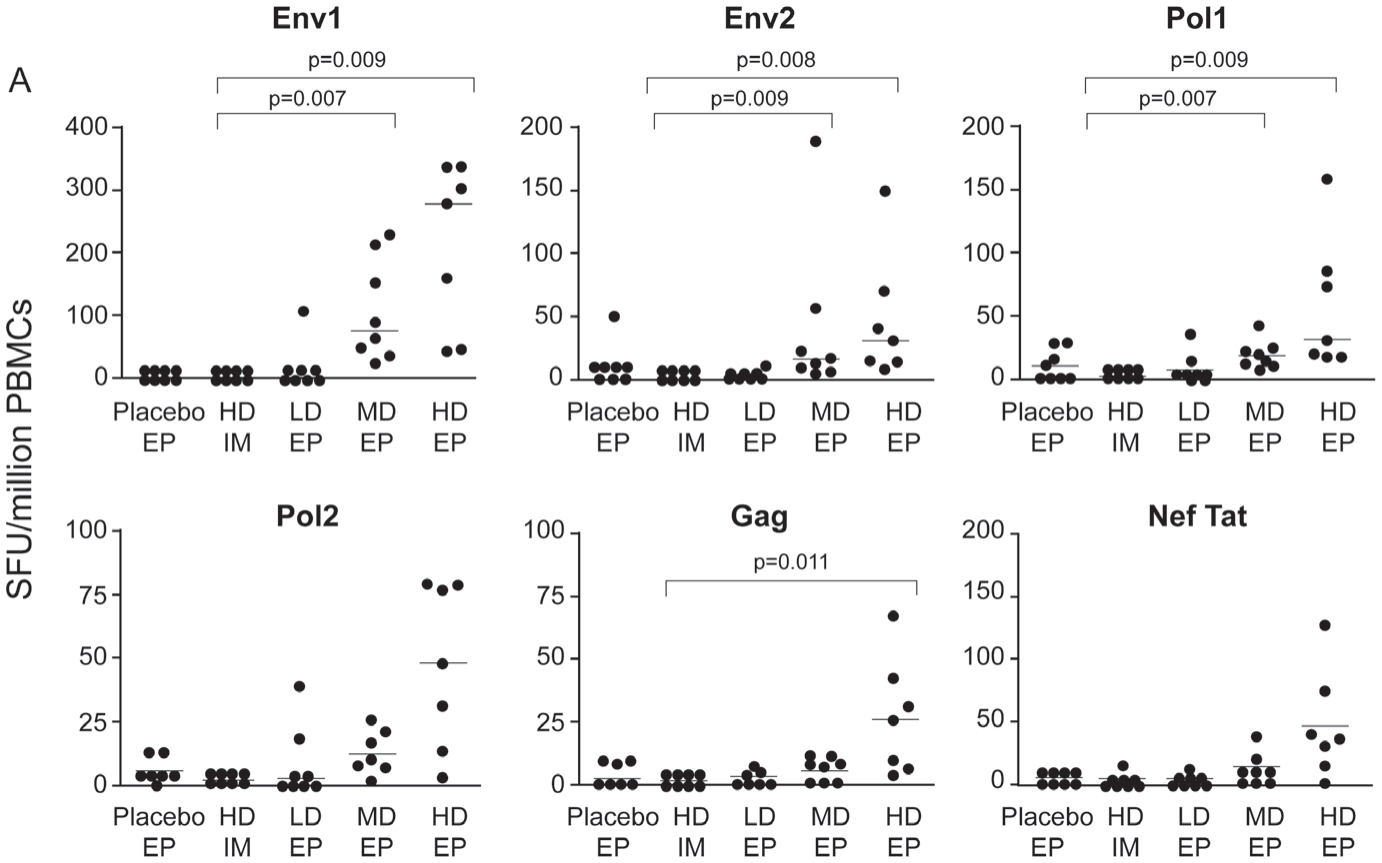
Individual IFNγ ELISpot Responses. All individual background-subtracted IFNγ ELISpot counts to each antigen at study Week 10, the peak response after the second administration. Horizontal lines indicate median values for each group. P-values indicate pair-wise comparisons of the three EP responses with HD-IM responses using the non-parametric Wilcoxon 2-sample test (t approximation). Significance is set at p<0.017, since there are 3 tests per antigen. Significant p values are depicted. SFU =  spot forming units.

Figure 4 depicts all individual background-subtracted IFNγ ELISpot counts for each peptide pool at Week 10. One volunteer in the HD-EP group missed the Week 10 visit, but completed all subsequent study visits. For each antigen, the magnitude of the background-subtracted count in the MD-EP and HD-EP groups tended to be higher than in the HD-IM group, although only statistically significant (p<.017) p-values are depicted on the graph. The difference in background-subtracted SFU between the LD-EP and HD-IM groups and between the Placebo-EP and HD-IM groups was not statistically significant.

Phenotypic analyses of the HD-EP T-cell responses at the time of peak IFNγ ELISpot response after the third vaccination are summarized in Figure S1. Seven of 8 volunteers mounted responses detectable by ICS, of which 4 formed CD3+ CD4+ responses alone, 2 formed both CD3+ CD4+ and CD3+ CD8+ responses, and one formed only a CD3+ CD8+ T cell response. The majority of responses were to Env, although 5/7 (71%) were to more than one antigen. Figure S1 B indicates the distribution of IFNγ, IL-2, MIP1β, and TNFα co-secretion in the CD3+ CD4+ and CD3+ CD8+ T cell compartments. The majority of responding cells in both the CD4+ and CD8+ compartments expressed a CD45RO+, CD27+ phenotype.

### Humoral Immunogenicity

Only one volunteer in the HD-IM group developed weak binding antibodies to clade C gp120 at 1∶50 serum dilution one week following vaccination that was sustained until week 8, after which time he was lost to follow up. All other responses in all volunteers at all time points were negative. One volunteer in the HD-EP group tested positive on HIV-1 ELISA at Week 56 with a simultaneous indeterminate western blot expressing a single gp160 band. A follow up test eight weeks later was negative for HIV-1 ELISA, western blot, and RNA-PCR (undetectable at <50 copies/mL).

## Discussion

This is the first demonstration that *in vivo* EP delivery of a DNA vaccine is safe, tolerable and acceptable to healthy volunteers. The level of tolerability was independent of age, gender, body weight, skin fold thickness, handedness, or sequence of vaccination, implying that such a technique could be evaluated on a wider population scale.

Table 3 and Figure 3 demonstrate that EP significantly improves the cellular immune response rate, magnitude, duration, and breadth of response to multiple antigens, consistent with previous results demonstrating the improved effect of EP in animal models and in humans with cancer [33]–[35]. Vaccination with the same 4 mg dose with and without EP increased the cellular immune response rate from 0 to 88%. As indicated in Figure 3A, the predominant responses were directed against Env, which may be due to differences in expression of the various genes in ADVAX, or due to a natural immunodominance. HIV-1 Env has been shown to induce preferentially higher immune responses in humans vaccinated with a multigenic viral-vectored vaccine [38].

The average magnitude of the anti-Env response in the HD-EP recipients was 70-fold higher than the mean response to the same antigen at the same dose delivered by standard IM injection (HD-IM). There was also a 22, 13, and 19 fold increase in the mean IFNγ ELIspot response to Pol, Gag, and Nef-Tat, respectively, a significant improvements over the 2–6 fold increases in cellular immunity to DNA vaccines afforded by cytokine adjuvants such as IL-12 and/or IL-15 in non-human primate studies [39]. EP also provided a dose-sparing effect, as the 1 mg dose also improved the immune response rate over the 4 mg IM vaccination. In addition, as shown in Figure 3B, these responses were durable, persisting through the end of the study, and broad, directed to multiple genes expressed by the vaccine. It has been well-documented that immune reponses to vaccines decrease with age [40]. It is therefore encouraging that the magnitude of ELISPOT responses did not wane with age, and were well-distributed among the volunteers, aged 18–59.

Phenotyping of these responses by ICS demonstrated that this T cell response tended to be a CD4+ T cell response, although a balanced CD4/8 response could also be detected in 29% of samples tested. The ability to elicit a strong CD4+ T cell response is one characteristic of DNA vaccines, in comparison to some viral vectors, which tend to elicit a predominantly CD8+ effector response [41]. EP delivery also improved the quality of the T-cell response, by inducing parallel secretion of IFNγ, IL-2, TNFα, and MIP1β in response to multiple antigens (Figure S1). These qualities have been associated with long-term improved control of HIV-1 infection and vaccine-induced protection from simian immunodeficiency virus (SIV) in monkeys [42], [43], although correlates of protection required for an effective HIV-1 vaccine remain unknown [44].

There is a wealth of data in animals demonstrating the ability of EP to improve the magnitude, duration, and quality of the humoral response to DNA vaccines [18]–[30], including preliminary reports in humans [35]. The low humoral responses in this study were likely due to characteristics of ADVAX, rather than ineffectiveness of the EP procedure, given the fact that ADVAX was initially designed to prime cellular immune responses to a matched modified Vaccinia Ankara (MVA)-based viral vaccine [36], rather than elicit humoral immunogenicity. ADAVX did not elicit a humoral response in humans after 3 IM vaccinations in a previous Phase 1 clinical trial [37].

In addition to these immunological advantages, DNA vaccines confer practical advantages for large scale global preventive vaccine campaigns, including the ability for repeated vaccination, relatively low cost and ease of manufacture, and favorable stability profile, even at higher temperatures. This report demonstrates that stand alone DNA vaccine regimens can elicit robust cellular immunogenicity in a dose-dependent manner when delivered by *in vivo* EP. In the future, this immunogenicity could be further enhanced by improving DNA vector design, delivering DNA at higher concentrations, with repeated administrations, and in conjunction with adjuvants. EP delivery of DNA vaccines may also improve priming before boosting with viral-vectored or protein vaccines. In parallel, electroporation devices are being re-engineered to be smaller and more portable. Thus, DNA-EP vaccine strategies may prove to be a promising approach to the prevention and/or treatment of multiple diseases, not limited to HIV-1.

## Supporting Information

Figure S1 **Phenotypic Analysis of Antigen-Specific T Cell Responses.** ELISpot responders from the high dose EP group were characterized by intracellular cytokine staining (ICS) as described in Methods. Panel A represents the distribution of CD3+ CD4+ and CD3+ CD8+ T cell responses. Panel B depicts the polyfunctionality of the antigen-specific response in each T cell compartment to all antigens, as assessed by co-secretion of IFNγ, IL-2, TNFα, and/or MIP1β.
(TIF)

Table S1 **Summary of Adverse Events by MedDRA System organ Class (SOC) and Dose Group.** Number of volunteers experiencing at least one adverse event in each SOC.
(DOC)

Protocol S1 **Trial protocol.**
(DOC)

Checklist S1 **CONSORT checklist.**
(DOCX)
