# Supplementary figures and images for: In Vivo Electroporation Enhances the Immunogenicity of an HIV-1 DNA Vaccine Candidate in Healthy Volunteers
Source: PLoS One. 2011 May 16;6(5):e19252. doi: 10.1371/journal.pone.0019252 (PMC3095594; doi:10.1371/journal.pone.0019252)

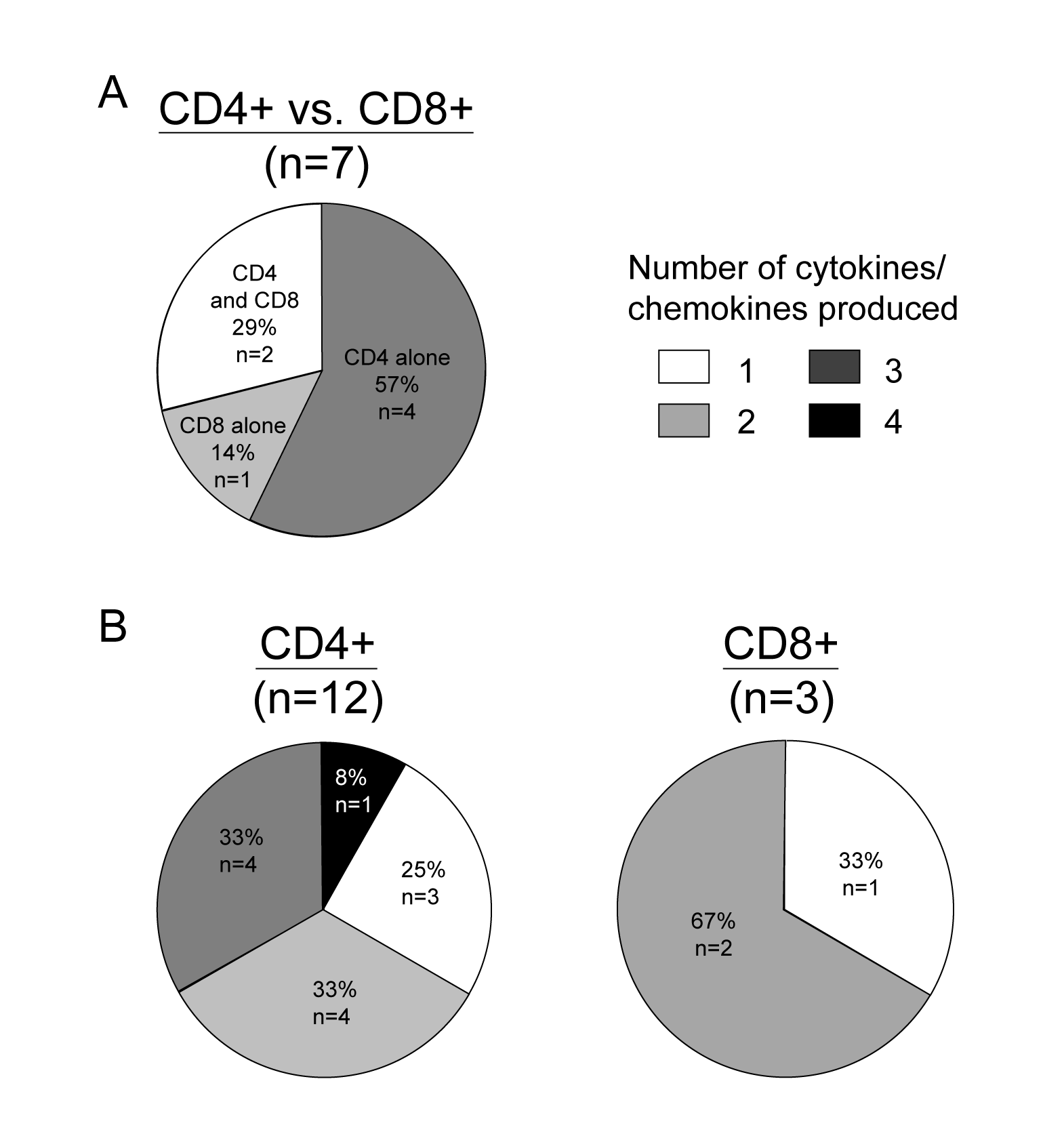

Supplement: Figure S1 — Phenotypic Analysis of Antigen-Specific T Cell Responses. ELISpot responders from the high dose EP group were characterized by intracellular cytokine staining (ICS) as described in Methods. Panel A represents the distribution of CD3+ CD4+ and CD3+ CD8+ T cell responses. Panel B depicts the polyfunctionality of the antigen-specific response in each T cell compartment to all antigens, as assessed by co-secretion of IFNγ, IL-2, TNFα, and/or MIP1β. (TIF) [file pone.0019252.s001.tif]
