# Supplementary material for: In Vivo Electroporation Enhances the Immunogenicity of an HIV-1 DNA Vaccine Candidate in Healthy Volunteers
Source: PLoS One. 2011 May 16;6(5):e19252. doi: 10.1371/journal.pone.0019252 (PMC3095594; doi:10.1371/journal.pone.0019252)
Supplement: Table S1 — Summary of Adverse Events by MedDRA System organ Class (SOC) and Dose Group. Number of volunteers experiencing at least one adverse event in each SOC. (DOC) [file pone.0019252.s002.doc]

**Table S1 – Summary of Adverse Events by MedDRA System organ Class (SOC) and Dose Group**

Number of volunteers experiencing at least one adverse event in each SOC

|  | Total (N=40) | Not related | | | | | | | | | | | Related* | |
| --- | --- | --- | --- | --- | --- | --- | --- | --- | --- | --- | --- | --- | --- | --- |
|  |  | EP 0.2 mg (N=8) | | EP 1.0 mg (N=8) | | EP 4.0 mg (N=8) | | IM 4 mg (N=8) | | Placebo (N=8) | | | EP 0.2 mg (N=8) | EP 4.0 mg (N=8) |
|  |  | Mild | Mod | Mild | Mod | Mild | Mod | Mild | Mod | Mild | Mod | Severe | Mild | Mild |
| Blood and lymphatic system disorders | 4 | 1 | 0 | 0 | 0 | 1 | 0 | 1 | 0 | 1 | 0 | 0 | 0 | 0 |
| Eye disorders | 1 | 0 | 0 | 0 | 0 | 1 | 0 | 0 | 0 | 0 | 0 | 0 | 0 | 0 |
| Gastrointestinal disorders | 4 | 1 | 0 | 0 | 1 | 0 | 0 | 1 | 0 | 1 | 0 | 0 | 0 | 0 |
| General disorders and administration site conditions | 6 | 1 | 0 | 0 | 0 | 1 | 0 | 1 | 0 | 1 | 0 | 1 | 0 | 1 |
| Immune system disorders | 1 | 0 | 1 | 0 | 0 | 0 | 0 | 0 | 0 | 0 | 0 | 0 | 0 | 0 |
| Infections and infestations | 8 | 1 | 1 | 1 | 1 | 1 | 0 | 1 | 1 | 1 | 0 | 0 | 0 | 0 |
| Injury, poisoning and procedural complications | 6 | 1 | 1 | 1 | 0 | 0 | 0 | 1 | 0 | 1 | 1 | 0 | 0 | 0 |
| Metabolism and nutrition disorders | 1 | 0 | 0 | 0 | 0 | 0 | 0 | 0 | 0 | 1 | 0 | 0 | 0 | 0 |
| Musculoskeletal and connective tissue disorders | 8 | 1 | 1 | 0 | 1 | 1 | 1 | 1 | 0 | 1 | 1 | 0 | 0 | 0 |
| Nervous system disorders | 7 | 1 | 0 | 1 | 0 | 1 | 0 | 1 | 0 | 1 | 1 | 0 | 0 | 1 |
| Psychiatric disorders | 3 | 1 | 0 | 1 | 0 | 0 | 0 | 0 | 0 | 1 | 0 | 0 | 0 | 0 |
| Reproductive system and breast disorders | 2 | 0 | 0 | 0 | 0 | 1 | 0 | 0 | 0 | 1 | 0 | 0 | 0 | 0 |
| Respiratory, thoracic and mediastinal disorders | 4 | 0 | 0 | 1 | 0 | 1 | 0 | 0 | 0 | 1 | 1 | 0 | 0 | 0 |
| Skin and subcutaneous tissue disorders | 6 | 1 | 0 | 1 | 0 | 0 | 1 | 1 | 0 | 1 | 0 | 0 | 1 | 0 |
| Surgical and medical procedures | 1 | 0 | 0 | 0 | 1 | 0 | 0 | 0 | 0 | 0 | 0 | 0 | 0 | 0 |

* Related = possible, probably, or definitely related. All 3 related events were classed as possibly related.
