# Supplementary material for: In Vivo Electroporation Enhances the Immunogenicity of an HIV-1 DNA Vaccine Candidate in Healthy Volunteers
Source: PLoS One. 2011 May 16;6(5):e19252. doi: 10.1371/journal.pone.0019252 (PMC3095594; doi:10.1371/journal.pone.0019252)
Supplement: Protocol S1 — Trial protocol. (DOC) [file pone.0019252.s003.doc]

**Research Study Submission to the**

**Institutional Review Board of The Rockefeller University**

1. **Principal Investigator**: David Ho, M.D.
2. **Investigators Who Will Conduct the Study**: Sandhya Vasan, M.D.

Sarah Schlesinger M.D.

Marina Caskey M.D.

Arlene Hurley R.N.

Daniel Dugin

1. **Title of Study:** Evaluation of Local and Systemic Reactogenicity Following Serial Administration of ADVAX, a Clade C DNA Vaccine, ADVAX e/g + ADVAX p/n-t, by Ichor TriGrid™ *in vivo* Electroporation to HIV-Uninfected, Healthy Volunteers
2. **Grant(s) in Which This Study is Described**: Bill & Melinda Gates Foundation Grant# 38648 Harnessing Dendritic Cells & Innate Immune Activation Signals to Guide HIV-1 Vaccine Development (PI, Ho) 07/01/06-06/30/11

1. **Type of Study**: Phase I Pilot study
2. **Date of Study**: October 2007
3. **Other Institutions Involved**: None
4. **Investigational New Drugs Involved**: ADVAX (ADVAX e/g + ADVAX p/n-t), a Clade C DNA vaccine. The IND number assigned to ADVAX by the FDA is BB-IND 11316. The IND number assigned to a current clinical trial using the TriGrid™ electroporation device is BB-IND-13275. The Device Master File assigned to the TriGrid^TM^ Electroporation device is MAF-1461. The IND number for this study is BB-IND-13477
5. **Radioactive Isotopes Involved**: None
6. **Rationale of Study, Research Plan and Procedures**.
7. **Abstract:**

HIV vaccines are desperately needed to slow or prevent the global spread of HIV. DNA vaccines encoding HIV antigens elicit cellular and humoral immunogenicity in animals, but are weakly immunogenic in humans. ADVAX, a Clade C/B' multigenic HIV candidate vaccine, has been shown to be both safe and immunogenic in healthy volunteers when administered intramuscularly in a Phase I dose-escalating clinical trial (DHO-519). ADVAX and other DNA vaccines are often intended for use in prime-boost strategies with viral vectors. However, issues with the manufacturing, scalability, storage, and distribution of viral vectors can complicate widespread global use. Conversely, DNA vaccines are easier and cheaper to manufacture, and do not require an intact cold chain for distribution, thus making them more suitable for developing world applications.

*In vivo* electroporation with the TriGrid^TM^ delivery system by Ichor Medical Systems, Inc. significantly enhances the cellular and humoral immunogenicity of DNA vaccines in animals, and has entered human clinical testing in cancer patients. This trial will investigate whether delivery of ADVAX by electroporation enhances either humoral or cellular immunogenicity in healthy volunteers. An enhanced immune response in the setting of electroporation could provide the proof-of-concept that DNA vaccines may act as stand alone vaccines against HIV or other diseases.

**Rational of Study, Research Plan and Procedures:**

This study is a Phase I, placebo controlled clinical trial to evaluate the administration of an HIV-1 vaccine encoding the *gag, env, pol, nef,* and *tat* antigens (ADVAX) in healthy volunteers by either *in vivo* electroporation or conventional intramuscular injection. The objectives of the study are to characterize the safety and tolerability of electroporation-mediated intramuscular delivery using the TriGrid^TM^ device by Ichor Medical Systems, Inc. and to compare the resulting antigen-specific immune responses to those elicited by conventional intramuscular injection of the vaccine. Safety of the vaccine delivered by electroporation will be characterized by documenting the nature, frequency, and severity of any toxicity associated with vaccination of ADVAX delivered by electroporation. Tolerability of the procedure will be assessed through the use of a brief questionnaire provided to the participants. Characterization of the immunogenicity of ADVAX delivered by electroporation or conventional intramuscular injection will include measurement of antigen-specific cellular and humoral immune responses. Recent data from ADVAX administered IM versus EP in Balb-C mice suggests that three vaccinations by EP elicit superior humoral and cellular immunogenicity than two vaccinations by EP. Two vaccinations have been safe and tolerable in all dose groups in humans to date. We have therefore secured enough vaccine to provide one additional vaccination to the high dose EP group at Week 36. This will allow participants in the high dose group who have been randomized to receive EP (either ADVAX or placebo) to elect to receive three vaccinations on a voluntary basis in order to maximize potential immunogenicity.

Study Procedures:

**Pre-Screening Questionnaire**

Potential participants will first undergo pre-screening by telephone to assess medical history and qualification for the study. They will have the opportunity to discuss the study and ask questions of the study recruiter at this time. Those who are eligible and interested in participation will attend a screening visit at the Rockefeller Hospital Outpatient Clinic. This is the same procedure that was conducted for the ADVAX (DHO-519) and ADMVA (DHO-549) trials.

**Screening Visit**

During the screening visit, study personnel will answer any questions about the study. Written site-specific informed consent will be obtained prior to conducting any study procedures. To ensure informed consent, the principal investigator or designee will discuss the following processes and explanations individually with each volunteer:

1. Pre HIV-test counseling
2. Risk-reduction counseling including safe-sex counseling
3. That it is unknown whether or not the study vaccine will protect against HIV infection or disease and, if so, the extent of that protection
4. That, following vaccination, it may be possible that the volunteer will develop antibodies against HIV, which may produce a positive reaction in a routine HIV test, and that provisions have been made to distinguish between response to vaccine and HIV infection during and after the trial
5. That a sexually active volunteer should use a reliable form of contraception from screening until end of study
6. Describe the electroporation procedure, including measurement of skin-fold thickness, both verbally and with a video.

If the volunteer consents to participate, site personnel will:

- Perform HIV risk assessment
- Perform complete medical history (including concomitant medication)
- Perform a general physical examination including height, weight, vital signs (pulse, respiratory rate, blood pressure and temperature), examination of skin, respiratory, cardiovascular and abdominal systems, and an assessment of cervical and axillary lymph nodes
- Perform a skin-fold measurement of the upper arm (Appendix B)
- Collect blood and urine specimens for all tests as indicated in the Schedule of Procedures (Appendix A)
- Perform a pregnancy test for all female volunteers

Screening laboratory test(s) may be repeated once at the discretion of the principal investigator or designee to investigate any isolated abnormalities.

If the screening visit occurs more than 42 days prior to date of vaccination, then study procedures for the screening visit must be repeated. However, the complete medical history may be replaced by an interim medical history and the informed consent form may be reviewed without signing again.

**Vaccination Visits (Day 0 and Week 8; and Week 36 for high dose volunteers in the EP group who elect to receive a third vaccination)**

Prior to the vaccination, site personnel will:

- Answer any questions about the study
- Review interim medical history (including concomitant medications)
- Review safety laboratory data
- Review the informed consent form administered at screening visit with volunteer
- Perform a directed physical examination including vital signs (pulse, respiratory rate, blood pressure and temperature) as well as an assessment of axillary lymph nodes and any further examination indicated by history or observation
- Conduct pre HIV-test counseling
- Perform a skin-fold measurement of the upper arm (Appendix B)
- Collect blood and urine specimens for all tests as indicated in the Schedule of Procedures (Appendix A)
- Perform a pregnancy test for all female volunteers (blood will be sent stat)and obtain results prior to vaccination
- Perform baseline assessment of the site of injection and evaluate and record any systemic symptoms
- Administer vaccine (the preferred site of administration is the deltoid muscle of the upper arm) either by standard intramuscular injection or by electroporation. Depending upon the amount of adipose tissue at injection site, the length and gauge of the standard intramuscular needle and the gauge of the electroporation device will be adjusted (Appendix B)

Volunteers will be closely observed for at least 30 – 45 minutes after vaccination. Vital signs (pulse, respiratory rate, blood pressure and temperature) will be monitored at 30 – 45 minutes after vaccination and recorded. Any local and systemic reactogenicity events, as well as any other event that occurs, will be recorded at 30 – 45 minutes. At this time, subjects will also be asked to complete a Vaccine Tolerability Assessment Form (Appendix D). Furthermore, volunteers will be given a diary card (Appendix E) and asked to record any reactogenicity events that occur between Day 1 and Day 7 and Day 57 and Day 64, as well as Day 253 and Day 260 in those volunteers in the high dose EP group who elect to receive a third vaccination. Site staff will explain to the volunteer how to record reactogenicity events.

**Post-Vaccination Visits**

Volunteers will receive a phone call from the study staff between 1 and 4 days after each vaccination and volunteers will be asked to return to the clinic 1, 2 and 4 weeks after the first vaccination (given at Day 0) and at weeks 9, 10 and 12 after the second vaccination (given at Week 8), and for those volunteers in the high dose EP group who elect to receive a third vaccination (given at Week 38) at Weeks 37, 38, 40, and 44. At those visits, the following will be conducted:

- Review of interim medical history and use of concomitant medications
- If symptoms are present, perform a symptom-directed physical examination
- Assess local and systemic reactogenicity as well as any other adverse events (At Week 44 only adverse events will be assessed)
- Collect blood and urine specimens for all tests as indicated in the Schedule of Procedures (Appendix A)

**Follow Up Visits**

At Weeks 16, 24, 36, 48, and 56, volunteers will be asked to return to the clinic for additional assessments of safety and immunogenicity. The following will be conducted at these visits:

- Review of interim medical history and use of concomitant medications
- If symptoms are present, perform a symptom-directed physical examination
- Assess any adverse events
- Collect blood and urine specimens for all tests as indicated in the Schedule of Procedures (Appendix A)

**a) Overview Introduction and Background**

In June 1981, the Centre for Disease Control (CDC) in the United States reported the first clinical evidence of a disease that would become known as Acquired Immunodeficiency Syndrome (AIDS). Twenty six years later, the AIDS epidemic has spread all over the world. Since the beginning of the epidemic, 65 million people have been infected.^1^ Globally, over 40 million people are today living with HIV infection, with over 5 million new infections acquired annually in 2001. More than 25 million individuals have lost their lives to the disease; in 2001, 3 million people died of AIDS. More than 90% of new HIV infections occur in developing countries, with the majority of infections found in Sub-Saharan Africa and South East Asia^1^. There is an urgent need to explore approaches to control the epidemic, in particular, preventive measures such as health education, treatment of sexually transmitted diseases, preventive vaccines and topical microbicides. In 2001, the Aaron Diamond AIDS Research Center partnered with the Rockefeller University and the International AIDS Vaccine Initiative (IAVI) to begin a program of HIV vaccine discovery and clinical development.

## DNA Vaccines

DNA vaccines contain a gene encoding one or more antigens under the regulation of a eukaryotic enhancer/promoter and polyadenylation signals that confer appropriate expression of the antigens. DNA encoding a selected component of a pathogen is injected as a plasmid. The identity of the immunogen gene is determined by sequencing. When injected into muscle, cells surrounding the injection site internalize the plasmid and transport the DNA to the nucleus. Transcription, translation and post-translational modification occur as they would in natural infection. The feasibility of genetic vaccination has been shown in several experimental model systems.^2-4^ Nucleic acid vaccines may elicit both antibody and CTL responses.^4-8^

The candidate DNA HIV vaccine ADVAX (ADVAX e/g + ADVAX p/n-t)

ADVAX is a DNA vaccine consisting of a mixture of two vectors (ADVAX e/g and ADVAX p/n-t) based on pVAX1 backbone, a commercially-available plasmid from Invitrogen®. pVAX1 was designed specifically for use in the development of DNA vaccines, and was constructed to be consistent with United States Food and Drug Administration (FDA) guidelines.^19^ It was modified by inserting an additional human elongation factor 1α (hEF1α) promoter. This alteration of pVAX1, yielding pADVAX, permits independent, high-level expression of a second genetic insert.

One of the vectors (ADVAX e/g) contains HIV-1 *env* and *gag* that have been modified for optimal mammalian expression. Codon optimization represents a facilitation of Rev/RRE-independent nuclear export,^9,10^ and is consistently found to enhance expression of viral genes. Overlapping PCR was used to unite oligonucleotides (80- to 90-mers overlapping by 16-18) with sequences reflecting this ideal codon selection. Gene expression was enhanced by 100- to 1000-fold as measured by ELISA or Western blot. Genes were further modified by incorporating a tissue plasminogen activator (tPA) leader sequence (amino acids: MDAMKRGLCCVLLLCGAVFVSAR), replacing the native sequence of *env* and supplementing the *gag* gene. Of note, this sequence is thought to enhance expression in part by facilitating transport of protein from the endoplasmic reticulum (ER) to the Golgi apparatus.^11-14^ With this refinement, gene expression was further enhanced by 3- to 5-fold. The second vector (ADVAX p/n-t) was constructed along similar lines. Overlapping PCR was used to unite "codon-optimized" oligonucleotides for synthesis of *pol*, *nef* and *tat*. This time, however, additional measures were taken to ensure safety for *in vivo* use. To prevent polypeptide processing a deletion was made in the active site of protease (PR) in the *pol* gene. Additionally, a point mutation was also made in the active site of reverse transcriptase (RT). To be able to incorporate all three genes into a single pADVAX-based vector, a *nef-tat* fusion gene, was constructed by overlapping PCR. All genetic sequences were kept intact, thereby preserving all immunogenic epitopes in the resultant fusion protein. As before, we added a tPA leader sequence to both *pol* and *nef-tat*. The ADVAX vaccine, then, involves two dual-promoter vectors: ADVAX e/g, which expresses *env* and *gag*, and ADVAXp/n-t, which expresses *pol* and *nef-tat*.

All five HIV-1 genes carried by these two vectors have been evaluated thoroughly *in vitro* to ensure expressive potential and safety in the context of these unique plasmid vectors. ADVAX, a DNA- based vaccine was constructed to express five different HIV-1 genes: *env, gag, pol, nef* and *tat*, incorporating certain safety mutations. The genetic sequences for both vaccines are based on the predominant circulating recombinant form derived from Kunming, China, where the epidemic has a strong foothold.

### Preclinical Studies with ADVAX e/g+p/n-t

Studies to assess the immunogenicity of ADVAX e/g+ADVAX p/n-t have been performed in BALB/c mice. For the purpose of gauging cell-mediated immune (CMI) responses, an ELISPOT assay was used to detect antigen-specific gamma interferon secretion by mouse splenoctyes. When three doses of plasmid DNA vaccine were administered in 3-week intervals, dose escalation experiments reveal a dose-response effect. Both CD4+ and CD8+ T cell mediated responses were induced against epitopes of all five plasmid-encoded HIV-1 immunogens, i.e. Env, Gag, Pol, Nef and Tat.

Humoral immune responses were also observed when anti-Gag antibody titers were measured in mice immunized with ADVAX e/g alone. Serum samples from immunized mice also demonstrate an antibody response to Env by western blot analysis.

In an evaluation of several prime-boost regimens using ADVAX plasmid DNA and ADMVA candidate vaccines, the DNA prime + MVA boost regimen induced the strongest CMI responses to peptides representing epitopes expressed by the five HIV-1 transgenes.

Studies were conducted to assess the potential systemic toxicity and reactogenicity of ADVAX e/g and ADVAX p/n-t in New Zealand White rabbits using the intramuscular route of administration, the intended route for clinical studies. All pre-clinical studies have been conducted in compliance with current U.S. FDA Good Laboratory Practice (GLP) Regulations for Non-clinical Laboratory Studies (21 CFR Part 58) to support safety claims. A 98-day repeat dose toxicity study was conducted at dose levels of 4 mg, 1 mg or vehicle (PBS), dosing at 4-week intervals. Twenty rabbits, ten females and ten males, were treated at each dose level. A total of four doses were administered over a 3 month period, representing one additional dose beyond the proposed clinical dosing regimen of 0, 1, and 3 months. The 4 mg dose level represents the high clinical dose proposed in the initial Phase I trial. Routine safety parameters were measured, including mortality, body weight gain, food consumption, clinical observations, ophthalmoscopy, local reactogenicity, clinical chemistry, hematology, and gross and microscopic histopathology at necropsy. Microscopic histopathological assessment was limited to the high dose group, i.e. 4 mg and control group. Interim necropsy of half of the animals in each group occurred at 48 hours post final dose, while the balance of animals were considered a recovery group, with final necropsy at 2 weeks post final dose.

Based upon the results of this study, intramuscular administration of up to 4 x 4 mg doses of ADVAX e/g+ADVAX p/n-t plasmid DNA vaccine over a 98-day interval was well tolerated in laboratory animals with no test article-related findings in mortality, clinical signs of toxicity, body weights, body weight changes, food consumption, ophthalmology, clinical pathology or gross pathology.

All but 2 of 52 animals survived until their scheduled termination and there were no adverse clinical observations noted that were attributed to treatment with the plasmid DNA vaccine. One male rabbit (1 mg) was euthanized due to luxation in the lumbar region, the result of handling the animal after scheduled blood sampling. One early death (female, 4 mg dose group) occurred 7 days after the initial dose. Prior to death on SD 8, the animal exhibited diarrhea, vaginal discharge, loss of appetite and lost 606 grams of body weight in a 7 day interval. Loss of appetite in this animal was evident at the initiation of dosing. Dehydration caused by the pre-existing condition of mucoid enteritis, an enteropathy of unknown etiology affecting rabbits, was considered to be the probable cause of death in this animal, given that anorexia and mucoid diarrhea were immediately evident at the initiation of the study.

Microscopic histopathological evaluation of 37 tissues from every high dose and control animal indicated that all were within histologic limits of normalcy for laboratory rabbits of the age and strain used in the study.

A second GLP study was conducted to determine the tissue distribution of a single intramuscular injection of the plasmid DNA vaccine and evaluate the persistence of the plasmids in a selected panel of tissues over a period of 60 days post-injection. Body weight gains, clinical observations and food consumption were also evaluated in this study. A single intramuscular injection of 200µg of ADVAX e/g+ADVAX p/n-t was administered to thirty CD-1 mice, an outbred strain. Mice in the control group received an equivalent volume of vehicle (PBS). At 3, 30 and 60 days post-injection, a subset of treated and control mice were sacrificed and specified tissues were removed for DNA extraction. Specifically blood, gonads, brain, lung, heart, spleen, kidney, liver, bone marrow, lymph nodes and the quadriceps muscle at the injection site were harvested at necropsy. Extracted DNA from tissues was analyzed for the presence of plasmid DNA sequences using a sensitive, plasmid-specific, qualified PCR assay. The quantitative PCR assay utilized primer and probe sets specific for the human elongation factor 1 alpha promoter and human CMV promoter sequences found in ADVAX e/g and ADVAX p/n-t plasmids. The design of the GLP biodistribution study provides the data necessary to evaluate intramuscular administration of ADVAX plasmids for potential aberrant tissue distribution and to define the kinetic profile of persistence of the plasmids, particularly as it relates to the potential for dissemination of plasmid DNA to the germ line.

No treatment related changes in mortality, clinical signs of toxicity, body weights, body weight changes, or food consumption were observed in CD-1 mice treated with ADVAX e/g+ADVAX p/n-t administered intramuscularly in the 60-day study. Analysis of integration potential using quantitative Polymerase Chain Reaction (qPCR) assays targeting the human cytomegalovirus (CMV) promoter and human elongation factor promoter DNA sequences, in combination with field inversion gel electrophoresis purification, indicated no evidence of detectable plasmid DNA integration into host genomic DNA.

### Human trials with DNA vaccines

Several studies have been performed in humans with DNA vaccines other than HIV.DNA vaccines. Anti-malaria DNA vaccines administered intramuscularly induced some CTL and no antibody responses, and were well tolerated up to 2.5 mg per dose.^15,16^ A pilot study (Protocol 400-003-03) with an HIV env/rev DNA construct was conducted in HIV-uninfected volunteers at the National Institutes of Health (NIH) Clinical Centre, USA. Doses as high as 3 mg were given as a single injection. Adverse events were mild and infrequent and T-cell responses were observed.^17^

A Phase 1 clinical study (AVEG Protocol 031) with an HIV-1 gag/pol DNA vaccine was performed in 52 HIV-1 uninfected adult volunteers aged 18-60 years with low risk for HIV-1 infection. Doses as high as 3 mg at 0, 1, 3 and 6 months were given. This vaccine was able to induce antigen-specific proliferation and production of chemokines by PBMC of vaccinated individuals, *in vitro*. Preliminary data on this HIV-1 gag-pol DNA vaccine were presented by Tellez et al. ^18^

In early 2000, Merck began a human trial of a DNA vaccine using HIV gag with the intention to boost with recombinant adenovirus. Preliminary data have been presented documenting safety and immunogenicity (Emini, CROI IX, Seattle, Washington, 2002).

A candidate DNA.HIVA vaccine has undergone Phase 1 clinical trials through the collaboration of IAVI, the Medical Research Council in Oxford, UK and with the Kenya Aids Vaccine Initiative (KAVI) in Nairobi, Kenya. The DNA.HIVA vaccine was given to a total of 31 volunteers at either 100µg (n=6) or 500 µg (n=25) intramuscularly. It has been well tolerated and immunogenic. Local side effects observed in the UK trial were mild to moderate, occurred mostly on the first post-vaccination day and resolved by 48 hours. Systemic side effects were mild to moderate and resolved within 1-3 days. In the Kenya trial no local or systemic side effects have been observed. A Phase 1 study testing a prime-boost approach and enrolling 120 volunteers started at the beginning of April 2002 in the UK. Dosage levels up to 2 mg DNA. HIVA per injection are being tested. As of May 19, 2003, approximately 103 individuals have been enrolled. No pattern of risk has been found among reported events. Thus far the vaccine has been found to be generally safe and well tolerated.

Human Trials with ADARC Vaccines

ADVAX was tested in a Phase I, randomized, placebo-controlled, dose escalating trial (DHO-519) at the Rockefeller University and the University of Rochester beginning December 2003. The trial was completed in June 2005 with no vaccine related serious adverse events. In addition, ADVAX elicited multigenic cellular immune responses, as measured by IFN-gamma ELISPOT, in volunteers at all dose groups.

ADMVA was tested in a separate Phase I, randomized, placebo-controlled, dose-escalating trial (DHO-549) at the Rockefeller University and the University of Rochester beginning December 2004. The last volunteer in this trial will be seen in mid July, 2007. To date, no vaccine-related serious adverse events have been reported. Preliminary testing indicates this vaccine elicits broad cellular and humoral immunogenicity.

One vaccination of ADMVA was administered to eight subjects who previously received ADVAX in a prime-boost Phase I pilot study (DHO-586) beginning in November 2006. The last volunteer visits were completed in June 2007, again with no vaccine related serious adverse events.

Rationale for the Use of the *In Vivo* Electroporation to Increase Immunogenicity

To date, the intramuscular administration of plasmid DNA vaccines in humans has been well-tolerated with few side effects. Although encouraging immune responses have been observed in some plasmid DNA vaccine trials, ^15-18^ the approach has generally been characterized by the inability to achieve effective and reproducible induction of the desired immune responses. This sub-optimal magnitude and consistency of response has been attributed to the poor transfection efficiency and low overall expression levels typically achieved following plasmid DNA administration in humans.^19^

Electroporation (EP) is a potent physical delivery technique based on the *in vivo* application of electrical fields that may facilitate plasmid DNA vaccination in humans (reviewed in 20) The EP technique utilizes propagation of electrical fields within a target region of tissue at a magnitude and duration sufficient to induce a transient increase in cell membrane permeability. During this transient state of cell membrane permeability, exogenous substances present in the interstitial space can be taken up into the cell. Shortly after pulse delivery the cell membrane function re-stabilizes and cells within the affected tissue resume normal function. The technique has been used extensively in clinical trials for the intratumoral delivery of bleomycin in a variety of tumor types.^20,21^ EP has also been shown in non-clinical studies to be a potent method for delivery of DNA plasmids. ^22-26^

EP has several important advantages that make it an appealing method for delivery of plasmid DNA vaccines. First, EP-mediated DNA delivery has been shown to induce both antibody and T cell responses.^23-26^ Second, EP is a non-viral delivery method. As such, it does not elicit unwanted immune responses against the vector. Thus, the technique is particularly appropriate for indications likely to require multiple vaccine administrations to achieve response. It is also advantageous because the absence of immunogenic viral antigens minimizes potentially deleterious antigen competition, thereby ensuring that the immune response is concentrated on the antigen of interest. Lastly, unlike other non-viral approaches, EP is capable of inducing consistent responses from subject to subject, even at relatively low DNA doses.^27^

Electroporation Administration System


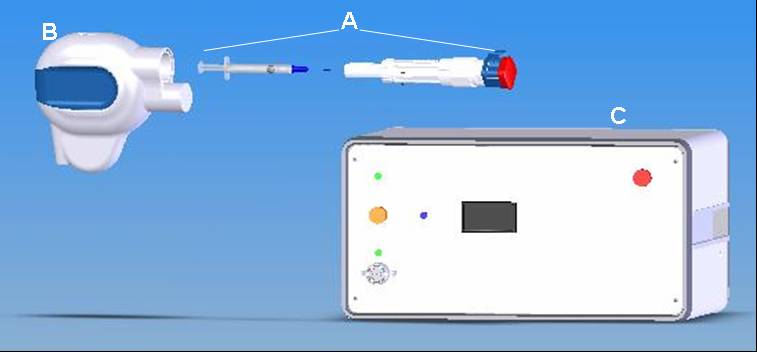


Ichor Medical Systems, Inc. has developed an EP administration system appropriate for intramuscular DNA delivery in the clinical setting. The EP delivery technique is dependent on the propagation of threshold level electrical fields at the site of DNA administration. This “co-localization” of electrical fields and therapeutic agent in the target tissue is a critical requirement for achieving efficacious DNA delivery. To ensure that the procedure can be applied consistently across a broad population of volunteers, Ichor has integrated the means for DNA delivery and electrical field propagation into a single, automated device. Thus, the Ichor system is designed for effective

and reproducible procedure application requiring minimal operator training. The Ichor system consists of three components (Figure): a single use Application Cartridge (A) an Integrated Applicator (B), and Pulse Stimulator (C).

The Application Cartridge is the only volunteer contact component. It encloses the four electrode TriGrid™ array and interfaces with a standard Becton Dickinson 3.0 cc syringe and 22 gauge 1.5 inch needle containing the agent for delivery. The Application Cartridge is packaged sterile for single use and is attached to the Integrated Applicator at the beginning of the procedure. The Application Cartridge also includes an automatic stick shield that locks over the electrodes and injection needle following procedure application. This allows safe and simple disposal of the single use applicator and insures that the electrodes and needle are never visible to the volunteer.

The Integrated Applicator is a reusable hand-held device that contains mechanisms to automatically deploy the electrodes and administer the biologic agent. The device is designed to allow the entire procedure to be applied in an automated fashion with the activation of a single button. This assures that the prescribed administration parameters will be implemented for every volunteer in a uniform fashion, thereby minimizing variability in the application of the procedure and reducing the need for operator training.

The Integrated Applicator is connected to the Pulse Stimulator through an incorporated cable. The Pulse Stimulator controls the administration sequence and generates the electrical signals necessary to enhance the intracellular delivery of the agent.

Safety Analysis of Electroporation Mediated Intramuscular Administration of ADVAX Plasmid DNA Vaccine in Rats – Assessment of Plasmid Biodistribution and Persistence and Integration

Overview: A study in small animals was performed to identify potential adverse events associated with EP mediated intramuscular delivery of a HIV-1 DNA vaccine (ADVAX) using the EP integrated applicator system for rodents (3.0 millimeters intraelectrode spacing). Specific endpoints included characterization of plasmid biodistribution and persistence / integration. The study was conducted in compliance with Good Laboratory Practices.

Methods: 100 micrograms of the Test Article was administered bilaterally in an injection volume of 25 microliters (total dose of 200 micrograms per subject) to the *tibialis* muscles of 28 rats (14 animals per sex) by EP (ADVAX + EP and 28 rats (14 animals per sex) by conventional direct injection (ADVAX Alone). Twelve negative control animals (6 rats per sex) were injected with Control Article (saline) by EP (Vehicle + EP).

At Day 7 after injection, 10 subjects in the ADVAX groups (5 per sex) and six subjects in the Vehicle Control group (3 per sex) were sacrificed. The presence of ADVAX plasmid in total DNA (genomic + plasmid) isolated from blood, bone marrow, heart, lungs, liver, spleen, kidney, brain, testis/ovaries, draining lymph nodes, muscles in which DNA was administered (bilateral tibialis anterior), and overlying skin from the injected muscle was examined. A real-time quantitative PCR (qPCR) assay for ADVAX plasmids with a Limit of Detection (LOD) of 10 copies / microgram genomic DNA and a Limit of Quantitation (LOQ) of 30 copies / microgram genomic DNA was used to estimate the levels of ADVAX in all tissues. At Day 60 the remaining subjects in each ADVAX group (9 per sex) and negative control group (3 per sex) were sacrificed and ADVAX levels were assessed for tissues and groups for which positive signals were observed at the Day 7 time point.

To assess the potential for integration of plasmid into genomic DNA, tissue samples from the Day 60 time point exhibiting > 1000 ADVAX copies / microgram genomic DNA were pooled with < 5 like tissue samples. Samples with low copy number (< 1000 copies / microgram genomic DNA) were excluded in order to reduce the potential for diluting plasmid signal. Pooled samples were then subjected to sequential agarose gel electrophoresis. This process is capable of separating high molecular weight genomic DNA from extrachromosomal ADVAX plasmid in order to assess the levels of ADVAX plasmid that co-purify with genomic DNA in a manner consistent with potential integration events.

Results:

Biodistribution: Quantitative PCR analyses for both administration methods indicated that, at all time points, ADVAX plasmid remained largely confined to the site of administration. At the Day 7 time point, 10 of 10 subjects in the ADVAX alone and 10 of 10 subjects in the ADVAX + EP group exhibited ADVAX presence in the skin and muscle samples. Detection of appreciable levels of plasmid outside of the site of administration following ADVAX delivery by EP was limited to the low level presence of ADVAX (< 1000 copies / microgram genomic DNA) detected in the lung sample from one of 10 subjects and in the draining lymph nodes of three of 10 subjects from the ADVAX + EP group. For the samples analyzed from the Day 60 time point, ADVAX plasmid was detected in the skin and muscle samples from the site of administration in 18 of 18 subjects in the ADVAX + EP and ADVAX Alone groups. The presence of ADVAX was not detected in any tissues outside of the administration site in any of the subjects from the Day 60 time point.

Plasmid Persistence / Integration: Analysis of genomic DNA samples from Day 7 samples in the ADVAX + EP group indicated initial ADVAX levels of ~5 x 10^4^ in the skin overlying the injected muscle and ~2 x 10^5^ copies / microgram in the injected muscle itself. By Day 60, ADVAX levels in the ADVAX + EP group had decreased by approximately 50 fold to ~8.1 x 10^2^ and ~5 x 10^3^ copies / microgram in skin and muscle samples respectively. This was comparable to the ADVAX levels observed in the skin and muscle samples from Day 60 in the ADVAX Alone group (~3.5 x 10^2^ and ~1.3 x 10^3^ copies / microgram respectively).

Based on these results, muscle and skin samples were analyzed to determine if the residual ADVAX exhibited behavior consistent with integration into the host genome. Four of 18 skin samples in the ADVAX + EP group and 2 of 18 skin samples in the ADVAX Alone group exhibited residual ADVAX levels of > 1000 copies. These samples were separated into two pools and one pool respectively. Sixteen of 18 muscle samples in the ADVAX + EP group and 11 of 18 samples in the ADVAX Alone group exhibited residual ADVAX levels of > 1000 copies. Samples from each group were separated into three pools each. Pooled samples were then subjected to sequential gel electrophoresis and analyzed for ADVAX content by qPCR. Following gel separation of the pooled DNA samples, all muscle and skin samples from both delivery methods (ADVAX Alone and ADVAX + EP) exhibited residual ADVAX plasmid levels below the LOQ for the assay (30 – 40 copies/ microgram genomic DNA depending on mass of DNA sample tested).

Key Findings / Conclusions:

• No significant adverse changes in general health status were detected in association with delivery of the ADVAX plasmid, either by EP or conventional intramuscular injection.

• The presence of appreciable levels of plasmid outside of the skin and muscle at the injection site following ADVAX administration by EP was limited to the low level presence of ADVAX detected in the draining lymph nodes in three of 10 subjects and the lung of one of ten subjects at the Day 7 time point.

• Following electrophoretic separation of plasmid and genomic DNA from tissue samples at the Day 60 time point, residual plasmid presence was below the Limit of Quantitation in the ADVAX + EP and ADVAX Alone groups .

• Under even the most conservative estimation, this level of plasmid integration represents a fraction of the estimated prevalence of spontaneously occurring gene inactivating mutations in the human genome.

Safety Analysis of Electroporation Mediated Intramuscular Administration of ADVAX Plasmid DNA Vaccine in Rabbits

In order to identify local and/or systemic adverse responses associated with the procedure, a general safety study in rabbits was undertaken after consultation with the US FDA. Animals were administered the Test Article at the highest DNA dose proposed for use in humans (4 mg in a 1 ml injection volume at 4.0 mg/mL concentration). Given that the proposed human trial will utilize two immunizations, the Test Article was administered three times during the safety study (at Study Days 0, 28, and 56) to 16 New Zealand White rabbits (8 male / 8 female) using Ichor’s clinical EP application system (ADVAX + EP). A group of 8 animals (5 male / 3 female) administered a sham procedure served as negative controls (Vehicle Controls). Animals were sedated for the administration procedure to facilitate procedure application and minimize startle reflex upon electrode/needle insertion and electrical field propagation. The Test Article was administered in the *vastus lateralis* muscle at the maximum DNA concentration and volume of injection proposed for use in humans (1.0 mL X 4.0 mg/mL). The *vastus lateralis* was selected as the administration site because its size and shape are reflective of the intended site of administration in humans, the medial deltoid. The EP administration procedure, electrode array composition and configuration as well as the specific electric field parameters were identical to those proposed for use in humans. Based on the proposed administration protocol in humans, the administration site was alternated between left and right *vastus lateralis* muscles on consecutive administrations.

Animals were monitored daily during the conduct of the study. A detailed evaluation of general health status (including physical exam and measurement of body mass) and assessment of the administration site was performed three times in the week following the Test Article administration and once weekly at other times. Observed abnormalities were documented as appropriate by narrative description and/or photograph. A blood chemistry panel and complete blood count (CBC) were performed on blood samples obtained within 72 hours after each Test Article administration. Where appropriate, a final blood sample was obtained prior to sacrifice.

Eight animals (4 male / 4 female) from the ADVAX + EP Group were sacrificed 48 hours following the final Test Article administration. Due to the anesthesia related early death of one subject (see Results section for discussion) the remaining cohort consisted of seven animals (4 male / 3 female) and was sacrificed two weeks after the final Test Article administration to assess near term recovery. Four animals from the naïve control group were sacrificed at each of the two time points (2 male and 2 female at the 48 hour time point and 3 males 1 female at the two week time point). The proposed sacrifice time points were selected to provide a relevant time frame for assessment of both the local tissue response as well as the development of potential toxicities arising in the weeks following the final Test Article administration. Following sacrifice, necropsy was performed including gross evaluation of organ systems and injection site. Histopathological analyses were performed on representative muscle and skin samples from the injection site as well as tissues recommended for assessment in repeat-dose toxicity studies by the Society of Toxicologic Pathology (6). Complete necropsy was performed for any unscheduled deaths in order to determine the cause of death.

Results:

In-Life Observations: Acute adverse events associated with intramuscular ADVAX administration with EP were limited to minor, transient cutaneous bleeding at the injection site in a minority of subjects. Occasionally, minor, transient ecchymosis was observed at the site of administration. The serial administration of ADVAX administration did not result in any discernable changes in body mass compared to Vehicle Controls. Of note, prior to the administration of the second ADVAX dose, one of the 16 subjects in the ADVAX + EP group exhibited a severe reaction (consisting of impaired respiration, reduced heart rate, and cyanosis) immediately following intravenous injection of the induction dose of a ketamine/xylazine cocktail. Attempts to resuscitate the animal were unsuccessful. Necropsy was performed and tissue samples obtained from the animal for pathological analysis. Hematology, blood chemistry, and general health data obtained from the animal until its death is included in the summaries below. Based on the results of these evaluations, the nature of the reaction, and its timing relative to anesthetic administration, it was concluded that the death was most likely attributable to the intravenous administration of the ketamine/xylazine cocktail used to anesthetize the subjects.

Blood Chemistry & Hematology:

Results from the analysis of blood samples obtained from subjects were analyzed based on reference values for rabbits provided in the Merck Veterinary Manual. Hematological abnormalities observed during the study were limited to low platelet counts observed in four animals during the study. Minor (< 10% below Lower Limit of Normal), transient decreases in platelet count were observed in two of 16 subjects receiving ADVAX + EP. More significant decreases (< 60% below Lower Limit of Normal) in platelet count were observed following in 2 of 8 Vehicle Controls. One subject in the ADVAX + EP group exhibited an elevated White Blood Cell Count following the first immunization.

Analysis of serum samples indicated that all Creatinine, Blood Urea Nitrogen (BUN), Aspartate aminotransferase (AST), Lactate dehydrogenase (LDH), and Alanine aminotransferase (ALT) readings were within normal reference ranges for all subjects at all time points. Alkaline Phosphatase (ALK) levels elevated above standard reference values were observed throughout the study in all subjects (including Vehicle controls) at all time points. No differences in the ALK levels were detected between any of the subjects, suggesting that the observed abnormalities were not due to EP application or ADVAX administration. Minor (< 2.5 times the Upper Limit of Normal), transient elevations in Creatine Phosphokinase (CPK) were observed in 3 of 16 subjects receiving ADVAX + EP and in 1 of 8 Vehicle Controls.

Sacrifice & Tissue Histology

Gross abnormalities noted during the sacrifice and tissue harvest were limited to minor injection site reactions. In the first cohort of the ADVAX + EP group (sacrificed two days after the third ADVAX immunization) the presence of minor hematomas in the muscle tissue from the recent (2 days prior) site of administration was noted in a majority of subjects. Gross evaluation of the contralateral site (in which ADVAX was delivered ~ 4 weeks prior) indicated the presence of focal interstitial myositis at the site of immunization. No abnormalities were noted in Vehicle Controls.

In the second cohort of the ADVAX + EP group (sacrificed two weeks after the third ADVAX immunization), abnormalities were limited to the presence of focal interstitial myositis at the sites of administration. The abnormalities were more pronounced in the more recent administration site (2 weeks prior) compared to the contralateral site (administration 6 weeks prior), suggesting ongoing resolution of the myositis. No significant gross abnormalities were noted in Vehicle Controls.

Microscopic analysis of tissue samples obtained from all subjects is currently underway.

Key Findings / Conclusions

• Acute adverse events associated with serial administration of ADVAX by EP were limited to minor cutaneous bleeding at the site of administration. Mild, transient bruising at the administration site was occasionally observed in the days following administration.

• No significant differences in general health status or body mass were observed in subjects administered ADVAX by EP compared to Vehicle Controls.

• Hematological and serum chemistry readouts were unremarkable. Abnormal values were rare, but when present were minor, transient, and typically observed in control subjects as well as ADVAX + EP subjects.

• Gross tissue abnormalities associated with ADVAX + EP were limited to injection site reactions.

Human Experience with Electroporation

Interest in EP for administration of DNA vaccines in the clinical setting is growing. Patient recruitment is currently underway for several therapeutic phase I trials of electroporation mediated plasmid DNA delivery, including one in the UK for prostate cancer patients and one multi-center international trial in colon cancer and breast cancer. The device proposed for use in this study (the TriGrid^TM^ Delivery System) is currently being tested in a Phase I study of a melanoma DNA vaccine at Memorial Sloan Kettering Cancer Center. All subjects have now completed two vaccinations, for a total of 64 administrations in 32 volunteers randomized to receive EP. There has been one Serious Adverse Event to date in a volunteer in the mid dose group (hospitalizations for chest pain/angioplasty/coronary artery bypass surgery) beginning at Week 24 that was deemed not related to vaccine.

**b) Hypothesis**

Delivery by electroporation will improve the immunogenicity of ADVAX in healthy volunteers.

**c) Objectives**

***Primary:***

- To evaluate the safety of an intramuscular prime and boost injection of the ADVAX DNA-based HIV vaccine via TriGrid™ electroporation at all three dosing levels

***Secondary:***

- To evaluate the immunogenicity of an intramuscular prime and boost injection of the ADVAX DNA-based HIV vaccine via TriGrid™ electroporation compared to placebo or standard syringe injection at all three dosing levels
- To evaluate the immunogenicity of an intramuscular prime and boost injection of the ADVAX DNA-based HIV vaccine via TriGrid™ electroporation at three dosage levels at all three dosing levels

**d) Primary Endpoints**

**Safety and Tolerability**:

- Severe local reactogenicity events (pain, tenderness, erythema/skin discoloration, edema, damage [vesiculation/ulceration], induration, formation of a crust or scab.
- Severe systemic reactogenicity events (fever, chills, headache, nausea, vomiting, malaise, myalgia, arthralgia, rash).
- Severe other adverse events (including laboratory abnormalities).
- All Serious Adverse Events.
- Proportion of volunteers with mild and moderate local and systemic reactogenicity events.
- Electroporation Tolerability Assessment Questionnaire Responses (Appendix B)

**e) Secondary Endpoints**

**Immunogenicity:**

Immunogenicity analyses will include:

- HIV-1 specific T-cell responses as quantified by IFNγ-ELISpot

- HIV-1 specific T-cell responses as quantified by multiparametric cytokine flow cytometry (CFC)
- Binding and neutralizing HIV antibody responses in blood and mucous secretions
- Additional studies as warranted at the discretion of the investigators.
- All immune responses will be evaluated for proportion of responders and the mean responses will be compared.

**f) Study Design**: The study is a Phase I pilot study that involves the administration of two injections of ADVAX vaccine or placebo, (with an optional third vaccination of ADVAX or placebo to the high dose EP cohort) that will be administered intramuscularly with or without electroporation to a total of forty healthy volunteers. The ADVAX vaccine with electroporation will be administered at three different dose groups, to eight individuals per dose group. ADVAX IM without electroporation will be administered at the highest dose (4mg) to volunteers. Eight volunteers will receive placebo. *An over-enrollment of 10% will be permitted.

| **Group** | **Dose** | **Material/Route** | **Subjects*** |
| --- | --- | --- | --- |
| **Group 1** | **0.2 mg** | **DNA /EP** | **8** |
| **Group 2** | **1 mg** | **DNA/EP** | **8** |
| **Group 3** | **4 mg** | **DNA/EP** | **8** |
| **Group 4** | **Saline** | **Saline/EP** | **8** |
| **Group 5** | **4 mg** | **IM** | **8** |

Safety and tolerability of the ADVAX vaccine will be evaluated after the two vaccinations given at Day 0 and Week 8 and at follow-up visits at 1, 2 ,4 weeks post the first vaccination and at weeks 9, 10 and 12 post second vaccination then at weeks 16, 24, 36, 48 and 56 post vaccinations. The eleven volunteers in the high dose EP cohort will be offered an optional third vaccination at week 36, with additional follow up visits at weeks 37, 38, 40, and 44.

Volunteers will be screened up to 42 days prior to enrollment and first vaccination and will be followed for 12 months after the second vaccination.

**Vaccination Schedule**

**Low and Mid Dose Groups, and High Dose IM Volunteers**

**Priming**

**Vaccination**

**Week 0**

**Week 8**

**Boosting**

**Vaccination**

**Week 56**

**Study**

**Conclusion**

**Priming**

**Vaccination**

**Boosting**

**Vaccination**

**Study**

**Follow-up**

**High Dose EP Group**

**Priming**

**Vaccination**

**Week 0**

**Week 36**

**Boosting**

**Vaccination**

**Week 56**

**Study**

**Conclusion**

**Priming**

**Optional Boosting**

**Vaccination**

**Study**

**Week 8**

**Follow-up**

**Administration Procedure**

ADVAX or placebo will be administered intramuscularly to the deltoid muscle. Subjects will be administered the study drug using Ichor Medical Systems’ intramuscular TriGrid™ delivery device. The Ichor device will be used only by personnel who have completed Ichor’s training seminar. The seminar will be conducted by Ichor personnel and will require approximately 60-90 minutes to complete. Topics will include setup and operation of the equipment, procedures for administration of the DNA vaccine, troubleshooting, and device care and storage. Each participant must successfully complete at least three mock administration procedures demonstrate competency.

Vials will be thawed at room temperature on the day of study visits. Immediately upon thawing and mixing of the vaccine vials, study vaccine/placebo will be withdrawn into 3.0 cc Becton Dickinson Model 309585 syringes under sterile conditions by the research pharmacist. Any unused portion of vials and syringes will be disposed of in accordance with Rockefeller University Pharmacy policy.

ADVAX will be administered as a single injection at the Day 0 and Week 8 time points, and for those volunteers in the high dose EP cohort who elect to receive a third vaccination, again at Week 36. The first and second vaccination will be given in alternating arms, and any optional third vaccinations will be given in the arm where the first vaccination was administered. Under no circumstances will the study vaccine/placebo be administered to regions of tissue that do not have intact lymph drainage. All subjects will be observed in clinic for a minimum of 30-45 minutes following each vaccination. Subjects receiving electroporation will complete a Tolerability Assessment Form (Appendix B).

**Storage and Shipment of the Investigational Vaccine**

The ADVAX e/g + ADVAX p/n-t vaccine and matched placebo will be shipped on dry ice from Fischer Scientific to the Rockefeller University Hospital Pharmacy where they will be stored at -30°C +/- 10°C.

**g) Statistical Model**

Randomization: will take place as indicated in the following table

| **Group** | | |  |
| --- | --- | --- | --- |
| **Placebo**  **(Saline/EP)** | **ADVAX IM** | **ADVAX/EP** | **DSMB evaluation prior to advancement** |
| **2** | **2 High Dose** | **8 Low Dose** | **12/12 subjects have received 1 vaccination**  **8/12 have received 2 vaccinations** |
| **3** | **3 High Dose** | **8 Mid Dose** | **14/14 subjects have received 1 vaccination**  **8/14 have received 2 vaccinations** |
| **3** | **3 High Dose** | **8 High Dose** |  |
| **8** | **8** | **24** | **Total Subjects** |

The randomization process will be performed by the Research Pharmacist at the Rockefeller University utilizing http://www.randomization.com.

The statistician at the Data Coordinating Center (EMMES Corporation), in collaboration with the principal investigators (or designees), will create tables according to a data analysis plan that has been reviewed and agreed to by the principal investigators (or designees). The EMMES Corporation will conduct the data analysis and will provide interim and final study reports for the principal investigators (or designees) and the regulatory authorities as appropriate. Prior to an analysis, additional monitoring visits will take place if necessary to validate the data held on the database, as well as all consent forms and dispensing records. Data files will be prepared by EMMES from a ‘frozen’ dataset for that particular analysis.

## Study Design

The study uses a dose-escalation design of an injection of the ADVAX DNA-based HIV via TriGrid™ electroporation compared to placebo or standard intramuscular injection. The study investigates three dose groups, 0.2, 1.0, and 4.0 mg. At the low dose (0.2 mg) stage, 12 volunteers randomized in a 2:2:8 ratio of placebo EP to ADVAX IM to ADVAX EP. At each of the mid and high dose stages, 14 volunteers will be enrolled in a 3:3:8 ratio of the corresponding treatment groups. All standard intramuscular injections will be high dose ADVAX vaccine.

## Sample Size and Power Calculations

A minimum of 40 volunteers will be enrolled in the study; 32 volunteers will be given active vaccines (24 EP and 8 IM) and 8 volunteers will be given placebo via EP route. An over-enrolment of about 10% (4 volunteers) will be permitted. This small sample size is appropriate for an exploratory dose-escalation study of a novel product while safety and immunogenicity of the vaccine are investigated.

The rate of Serious Adverse Events related to vaccine will be used as one measure of the safety of the candidate vaccine. Adverse Events that may be temporarily incapacitating (for example, loss or cancellation of work or social activities), which could make a vaccine impractical for large scale use if they occur in more than a small proportion of cases, will also be assessed.

One method of assessing the sample size is to consider the precision for estimation of incidence of serious adverse events.

*Prior to the enrollment of the medium dose level group:*

If none of the volunteers receiving the vaccine experiences an SAE related to vaccine (n=8), the 95% upper confidence bound for the rate of these adverse events in the population is 0.37 by Clopper Pearson method.

*Prior to the enrollment of the highest dose level group:*

If none of the volunteers receiving the vaccine experiences an SAE related to vaccine (n=16), the 95% upper confidence bound for the rate of these adverse events in the population is 0.21.

*After all volunteers have received the initial dose:*

If none of the volunteers receiving the vaccine experiences an SAE related to vaccine (n=24), the 95% upper confidence bound for the rate of these adverse events in the population is 0.14.

There is very limited power for comparison of event rates between treatment arms.

## Analysis

The analysis of study data will be primarily descriptive, with emphasis on tabular and graphical displays. Summary statistics will be calculated, along with point and interval estimates of solicited and unsolicited adverse event and immune response rates. This study is exploratory, and any statistical inferences will be hypothesis generating, and not confirming.

***Safety and Tolerability:***

All adverse events will be reported, grouped as to whether or not they qualify as SAEs, their severity grading, and their relationship to vaccine (as judged by the investigator and reviewed by the Safety Review Board).

***Vaccine Immunogenicity:***

Cellular immune responses will be analyzed using binomial methods to examine for the presence or absence of HIV-specific T-cell responses quantified by ELISPOT and cytokine flow cytometry (CFC). Presence or absence of anti-gp120 antibodies will be also analyzed in the blood. Assays will be performed in a similar fashion in all volunteers. Because of the small sample sizes and multiple epitopes, the results will be primarily descriptive.

Based on the previous experience with IAVI Phase I vaccine studies, it is expected that the amount of missing, unused or spurious data will be insignificant. Unused and spurious data will be listed separately and excluded from the statistical analysis. Missing data will be excluded from the statistical analysis. A data analysis plan will be developed and agreed upon by IAVI, ADARC, and the investigators prior to unblinding.

11. **Subjects of Study**: Healthy male and female subjects aged 18-60 who are not at high risk for acquiring infection with HIV-1 will be enrolled if they meet the following eligibility criteria:

High risk is defined by any of the following over the past 6 months:

- Unprotected vaginal or anal sex with a known HIV positive person or a casual partner (i.e. no continuing established relationship)
- Engaging in sex work for money or drugs
- Using injection drugs (illicit), or
- Acquiring a sexually transmitted disease in the past six months

**Inclusion Criteria:**

1. Healthy adult males and females, as assessed by a medical history, physical exam, and laboratory tests
2. Age of at least 18 years of age on the day of screening and no greater than 60 years at time of vaccination
3. Willing to comply with the requirements of the protocol and available for follow-up for the planned duration of the study (screening plus 14 months)
4. In the opinion of the principal investigator or designee, has understood the information provided. Written informed consent needs to be given before any study-related procedures are performed
5. Willing to undergo HIV Testing and counseling, and receive HIV test results
6. If sexually active female, using an effective method of contraception (combined oral contraceptive pill; injectable contraceptive; diaphragm; Intra Uterine Device (IUD); condoms; anatomical sterility in self or partner) throughout the study period. All female volunteers must be willing to undergo urine pregnancy tests at time points as indicated in the Schedule of Procedures (Appendix A)
7. If sexually active male, willing to use an effective method of contraception (such as condoms, anatomical sterility) throughout the study period and will be advised not to get his partner pregnant

**Exclusion Criteria:**

1. Confirmed HIV-1 or HIV-2 infection
2. Any clinically significant abnormality on history or examination including history of immunodeficiency or autoimmune disease; use of systemic corticosteroids immunosuppressive, anticancer, or other medications considered significant by the trial physician within the last 6 months
3. Any clinically significant acute or chronic medical condition requiring care of a physician (e.g., diabetes, coronary artery disease, rheumatologic illness, malignancy, substance abuse) that in the opinion of the investigator would preclude participation
4. Any laboratory value outside of reference range, with the exception of any non-clinically significant Grade I elevations of liver function tests (AST, ALT, direct/total bilirubin), CBC, and urinalysis, as determined by the Principal Investigator or his designee
5. Confirmed diagnosis of hepatitis B (surface antigen, HbsAg); hepatitis C (HCV antibodies) or active syphilis
6. If female, pregnant, planning a pregnancy during the trial period, or lactating
7. Receipt of a live attenuated vaccine (other than influenza) within 30 days or other vaccine within 14 days of ADVAX vaccination
8. Receipt of blood transfusion or blood products 6 months prior to vaccination
9. Participation in another clinical study of an investigational product currently or within past 12 weeks, or expected participation during this study
10. History of severe local or systemic reactogenicity to vaccination or history of severe allergic reactions
11. Major psychiatric illness including any history of schizophrenia or severe psychosis, bipolar disorder requiring therapy, suicidal attempt or ideation in the previous 3 years
12. Any electronic stimulation device, such as cardiac demand pacemakers, automatic implantable cardiac defibrillator, nerve stimulators, or deep brain stimulators
13. Individuals in which a skin-fold measurement of the cutaneous and subcutaneous tissue for all eligible injection sites (deltoid muscles with intact lymph drainage) exceeds 40 mm
14. In the opinion of the investigator, unlikely to comply with protocol

12. **Recruitment and Consent Procedures**:

Volunteers will be recruited via approved advertisements on the internet (e.g. [www.craigslist.org](http://www.craigslist.org/)), newspapers (e.g. AM New York, Metro), and flyers. Special precautions will be used in recruiting employees of Rockefeller University or the International AIDS Vaccine Initiative to minimize the possibility of coercion. Rockefeller University employees will be made aware of the study via campus-wide emails and flyers, rather than directed presentations to selected groups. Only individuals outside of the Ho and Steinman labs and non-hospital employees may be recruited. IAVI employees will not be recruited through presentations at the workplace. Only individuals outside of the clinical project team may be recruited. Subjects will be reassured that refusal to participate or early termination will not affect their studies or employment in any way, and that all medical records will be kept confidential in accordance with HIPAA guidelines.

Participants will be given a copy of the informed consent form at the time of their screening visit. Participants will also have the opportunity to watch a video describing the electroporation procedure. A member of the research team as listed above will be available to answer any and all questions regarding the conduct of this study. A signature at the end of the informed consent will indicate that the participant understood the nature of the research and agreed to participate.

12. **Risks and Alternative Methods**:

Participation in this study involves the following risks:

1. Blood will be taken from participants as indicated in the study procedures. Phlebotomy can cause temporary local pain, bruising, and, rarely, infection
2. Rarely, fainting during phlebotomy may occur
3. Following administration of the vaccine/placebo participants may experience pain, soreness, redness/discoloration and bruising around the site of the injection
4. Due to the insertion and activation of multiple electrodes, the use of electroporation may increase these risks
5. The application of electroporation will result in a brief, localized muscle contraction at the site of administration, which may be painful. Approximately 10% of subjects may experience a tingling sensation in their arm and fingers after administration. Since intramuscular DNA delivery with electroporation results in increased intracellular uptake of plasmid at the site of injection and electric field application, the procedure may increase the frequency and/or severity of local site reactions. Such symptoms should not last longer than several days. However, volunteers will be removed from the study if injection reactions ≥ Grade 3 in severity occur
6. We do not know what effect the vaccine would have on an unborn child if given to a pregnant woman. Females of childbearing potential will be advised to use a reliable form of contraception, as discussed with a nurse counselor, until study completion. A pregnancy test will be performed at screening, on the day of vaccination, and at every visit throughout the course of the fourteen-month study. Males who are not anatomically sterile (those who have had a vasectomy or medically proven to be sterile) should use condoms until study completion to avoid pregnancy in a spouse or partner
7. Following vaccination a participant may become HIV positive on a routine HIV test. This test result does not mean that person has HIV or AIDS. It could mean that exposure to the vaccine resulted in the production of antibodies to it. In case of a positive HIV test result, an independent laboratory will confirm whether the positive test is the result of the vaccination or whether the participant contracted HIV through exposure in the community. In the event that an individual has a vaccine-induced positive serology, ADARC will provide documentation of that individual’s participation in this vaccine trial
8. It is unknown whether receiving this HIV vaccine will alter the immune response to any future HIV vaccine that may be developed in the future
9. The possibility of integration of the DNA plasmid vector into genomic DNA of transfected myocytes has been considered. Plasmid integration at a sufficiently high frequency carries the possibility of inducing deleterious gene expression modifying mutations (e.g. inactivation of a tumor suppressor gene). Potential side effects could include an increased risk of malignancy arising from the cells harboring the mutation(s).

The enhanced intracellular uptake of plasmid DNA following intramuscular delivery with electroporation raises the prospect that this technique could increase the risk of plasmid integration. An analysis of ADVAX persistence / integration levels was conducted with the EP. The EP procedure resulted in a plasmid copy number associated with genomic DNA following electrophoretic purification of less than 40 plasmid copies per microgram of genomic DNA at Day 60.

Utilizing the Limit of Quantitation of the assay (40 plasmid copies / microgram of genomic DNA) as a baseline estimate of residual plasmid, and assuming that each of the plasmid copies induces a gene inactivating mutation, the frequency of induced mutation for any single gene following electroporation mediated DNA delivery can be conservatively estimated at 1 x 10^-8^ (i.e. 1 mutation per 10^8^ cells). Using this conservative estimate, the number of mutations introduced by the procedure would be two to three orders of magnitude lower than the prevalence of gene inactivating mutations detected in healthy humans for a number of model genes including hypoxanthine-guanine phosphoribosyltransferase (HPRT), ^35^ glycophorin A, ^36^ and the HLA A locus. ^37,38^

j. The theoretical possibility of uptake and integration of the DNA plasmid vector into tissues outside of the administration site has been considered. The most concerning scenario would be uptake and integration of plasmid into reproductive tissues, potentially leading to vertical transmission of the vector. However, the highly localized nature of the DNA injection and electric field application indicate that such an occurrence is extremely unlikely. The results of the vector biodistribution study further support this conclusion. No long term persistence of plasmid was detected in tissues outside of the injection site. No plasmid vector was detected in any animal’s gonadal tissues, suggesting minimal risk of vertical transmission of the plasmid.

k. There is a theoretical risk that an immune response may form to the non-HIV DNA in the vaccine, which could cross-react with autologous host DNA. This has never been reported in any DNA vaccine administered to humans to date. Nonetheless, we will monitor for the formation of anti-DS DNA antibodies at three timepoints during the course of this study.

14. **Data and Safety Monitoring Plan**:

1. **Risk:** This project entails **moderate risk** to subjects. Monitoring will be conducted a) by the Principal Investigator (PI) in consultation with the Clinical Research Officer of the Clinical Translation Science Advisory Committee and b) by the PI and the Rockefeller University Institutional Review Board (IRB), through annual reports of progress and by immediate notification of adverse events by the PI to the IRB, the ACCTS, and any federal regulatory agency (e.g. FDA) when appropriate or required.

2. **Determine the need for a DSMB:** This study is a Phase I study which exposes the subjects to “moderate risk” as the ADVAX vaccine is an experimental vaccine that will be administered using an experimental delivery device TriGrid™ that has never been administered to healthy volunteers before. A Data and Safety Monitoring Board (DSMB) will be established for this study. See details about the DSMB below.

3. **Safety Review Plan:** Subjects will have regularly scheduled visits to the outpatient clinic. Please see Appendix A “Schedule of Procedures” for the details of these visits. Safety laboratory testing (hematology, clinical chemistries, pregnancy testing, hepatitis B/C profiles, RPR/TPHA testing, anti-DS-DNA antibody test, and urinalysis) will be performed at the Memorial Sloan Kettering Cancer Center (MSKCC). The results of these tests will be reviewed, evaluated, signed and dated by the Principal Investigator or his designee in real time (for routine labs and CD4/8 cell counts, 24 to 48 hours from the time of the lab analysis). The results of the HIV antibody tests at the initial screening, performed by the New York State Department of Health AIDS Institute (NYS DOH AI), will be available for review and evaluation from 72 to 120 hours after lab analysis dependent upon the test result. After the trial has commenced, on all subsequent HIV antibody tests where the results are positive, the NYS DOH AI will also perform an HIV-RNA test on that sample. Other specialized virological and immunological testing will be performed either in real time or “batched” and analyzed at a later date. Abnormal laboratory results will be reviewed and discussed by the clinical team on a regular basis at the weekly clinical meeting at ADARC and minutes of those discussions will be kept. A copy of routine laboratory results from CLEP certified laboratories will be provided to each subject during their outpatient visits.

No more than one volunteer per day will be enrolled for the first four volunteers to receive electroporation in each dose group. For these four volunteers, an additional telephone call to assess safety and adverse events will be made on the day following vaccination. If four vaccinations by electroporation have occurred without greater than moderate adverse events, the remainder of the group can be enrolled with no fixed interval between volunteers.

A. **Description of Adverse Events (AE) Detection and Reporting:**

**i. Adverse Event Definition**: An adverse event is any noxious, pathologic, or unintended change in anatomical, physiologic, or metabolic functions, as indicated by physical signs, symptoms, and/or laboratory changes occurring in any phase of the clinical trial, whether associated with drug and whether or not considered drug related. All of the following are to be considered adverse events:

- An exacerbation of a pre-existing condition.
- An inter-current illness.
- Any drug interaction.
- Any event related to a concomitant medication.
- Development of an abnormal laboratory value or a significant change from baseline in a laboratory value within the range of normal, considered by the investigator to be clinically important.

Any adverse event prior to the first vaccination will be reported as a pre-existing condition on the “previous conditions” source document. At each outpatient visit, subjects will be questioned and/or examined by the PI or his designee for evidence of AEs. The onset and end dates, severity and relationship to study vaccine and EP device will be recorded in the medical record for each event.

**ii. Assessment of Severity:** The severity of the AE will be assessed according to the Adverse Event Severity Assessment Table (see Appendix C).

**iii. Serious Adverse Event (SAE) Definition:** An SAE is an AE that results in any of the following outcomes.

- Death
- Life threatening condition
- Hospitalization or prolongation of stay
- Persistent or significant disability or incapacity
- Congenital anomaly or birth defect
- Medically significant event, including laboratory abnormalities

Important medical events that do not result in death, are life threatening, or require hospitalization, may be considered SAEs when, based on appropriate medical judgment, they may jeopardize the volunteer, or may require medical/surgical intervention to prevent one of the outcomes listed in this definition.

A life threatening event means an immediate risk of death from the event as it occurred. Life threatening does not include an event that, had it occurred in a more serious form, might have caused death. For example; drug-induced hepatitis that resolved without evidence of hepatic failure would not be considered life threatening even though drug-induced hepatitis can be fatal.

Any serious adverse event (SAE) and unexpected adverse event involving human subjects of an IRB-RU approved protocol will be reported to the Rockefeller University IRB within two (2) working days of the discovery. These events includes serious adverse events that occur anytime while enrolled in the study or within 30 days after a subject completed the study if reported spontaneously to the study team. The date of study completion for any subject is defined as either 30 days following the last study visit or the date a participant and/or Investigator determines that the participant can no longer comply with the requirements for any further study visits and evaluations, (e.g., the participant is prematurely discontinued from the study). In addition to the adverse event reports to Rockefeller, these reports should be appropriately reported to FDA in compliance with 21 CFR 312.32.

B. **Unanticipated Adverse Event Reporting:** Other adverse events that require written reporting to the IRB-RU within five (5) working days of the discovery include: [1] Any unanticipated problems or deviations from protocol conduct involving or creating more than minimal risk to subjects or others, or irregularities in the process of informed consent; [2] Physical or emotional harm to the subject during the execution of the experimental protocol; [3] A breach of confidentiality or privacy and; [4] Unexpected harmful effects of an investigational or FDA-approved drug, biologic or device, observed in other research setting similar to that of the approved project.

C. **Annual Reporting of AEs:** All AEs will be summarized in tabular form for the annual review of the study by the IRB and ACCTS. Information relating to each AE will include the grade, the attribution to study drug or procedure and where applicable, any study drug dose reduction or discontinuation. Total enrollment, demographics of enrollment, protocol violations, current status of subjects (completed, ongoing, premature discontinuation) reasons for attrition, and number of subjects reaching primary endpoints and available research data, especially any that might provide evidence of early proof or disproof of the hypothesis, will also provided in the annual report.

4. **Data Management:** Principal Investigator will oversee how the data are collected, entered, and protected. Data will be entered into case report forms (CRFs) by the Study coordinator and/or Data Manager. Data generated at the immunology laboratories will be transferred directly to the Data Coordinating Center (The EMMES Corporation) as soon as feasible, e.g., within 2 weeks of the assay being performed. Data will be entered into the EMMES internet-based Data Entry System (IDES). Consistency checks and range checks will be performed by data entry and supervisory personnel. Queries raised by the monitor or by the EMMES Corporation will be directed to the Investigators and study staff. To ensure the quality and reliability of data gathered and the ethical conduct of this trial, standard operating procedures have been developed for all clinic and laboratory procedures. Regular monitoring will be performed according to Good Clinical Practice (GCP). All source documents will be kept in a locked facility at the clinical site. All medical records (when not being reviewed by the research team) will be kept under lock and key in the Medical Record Department of the hospital with access limited to the appropriate RUH personnel, ADARC personnel, members of the IRB and the FDA. The sponsor of the other two vaccine trials, now completed (DHO-519 and DHO-549), the Investigator the International AIDS Vaccine Initiative (IAVI) will provide a Clinical Research Associate (CRA) who will conduct scheduled monitoring visits and will have access to the subjects’ medical records.

5. **Study Monitoring:** The conduct of the study as well as the integrity of the data will be monitored carefully and closely. In accordance with applicable regulations and Good Clinical Practice, monitors will periodically contact the site, including conducting on-site visits. The monitor for this study will be a Clinical Research Associate employed by IAVI. Items for on-site review include meeting of eligibility requirements, correct execution of the informed consent process, evidence of the re-consenting process for updated informed consent forms, complete and eligible source document entries, meeting of visit and test schedules, adverse events (as described in detail above) reporting and evidence of appropriate action (as needed), and the appropriate documentation of changes to study vaccine dosing schedules, or suspension of the vaccinations. The extent, nature and frequency of on-site visits will be based on such considerations as the study objectives and/or endpoints, the purpose of the study, study design complexity, and enrollment rate.

6. **CLIA and CLEP Laboratory Certification:** MSKCC, ADARC and NYS DOH AI laboratories have New York State CLEP certification. Only results from certified laboratories will be communicated to the subjects, given to referring physician and used to influence a decision regarding the participant’s treatment and further participation in this study.

**A Data and Safety Monitoring Board has been composed to oversee data and safety for the Rockefeller/ADARC site.**

## Standard Operating Procedures (SOPs) for the Data and Safety Monitoring Board (DSMB):

- The charter of the DSMB is to provide an ongoing assessment of volunteer safety during the ADVAX + EP Clinical Trial. The DSMB will consist of independent individuals who have no relationship to the Principal Investigator and Co-Investigators involved in the trial. No member of the DSMB will have any direct responsibility for the clinical care of trial volunteers. No representative of IAVI, Ichor or ADARC or their designees may be a member of the DSMB. However, the DSMB may invite the principal investigator (PI) or designee and an IAVI and/or ADARC representative to an open session of a DSMB meeting to provide information on study conduct, present data, or to respond to the members’ questions.
- The names, university affiliation and title, area of expertise, and contact information of each of the DSMB members are provided to the IRB (as listed above).
- All updated versions of the protocol and Investigator Brochure (I.B.) will be provided to the DSMB members.
- The DSMB will teleconference to review the trial data at least 14 days after both of the following criteria have occurred in the first group: after 12 volunteers in the first group have received their first vaccination *and* at least 8 volunteers in the same group have received both vaccinations. Similarly for group two, the DSMB will teleconference after 14 volunteers in the second group have received their first vaccination *and* at least 8 volunteers in the same group have received both vaccinations before proceeding to the next dose group. The DSMB must approve escalation to the next dose. At least two members are required to review the data before approval. Additional meetings will take place if there are indications for an interim review, which may be unblinded at the discretion of the DSMB.
- The study team will provide the DSMB with updated records of all adverse events (AEs) of a grade 2 or higher prior to each teleconference. The DSMB will be asked to review all severe clinical adverse/reactogenicity events judged by the Principal Investigator or designee to be possibly, probably or definitely related to vaccine.
- The DSMB will be asked to review on an interim basis all severe laboratory adverse events confirmed on retest and judged by the Principal Investigator or designee to be possibly, probably, or definitely related to vaccine.
- The DSMB will provide a written report to the PI after each teleconference and the PI in turn will distribute these reports to the study team, the sponsor, and the IRB.

### Indications for discontinuation of vaccinations in all volunteers:

If 3 or more of the volunteers participating in this trial develop an SAE or severe AE judged as definitely, probably or possibly related to the vaccine, and clinically significant by the Principal Investigator or designee, IAVI will request a review by the DSMB and the trial will be suspended pending a review of all safety data by the DSMB. The study treatment may be unblinded at the discretion of the DSMB. Following this review, the DSMB will make a recommendation to the Principal Investigator and IAVI regarding the continuation of the trial. Unless the trial is permanently discontinued, PI, the research team, and IAVI will not be made aware of any unblinded results.

15. **Procedures to Minimize Risks**: All study procedures will be performed by David Ho, M.D. and his designees. All vaccinations with electroporation will be performed only by individuals who have successfully completed Ichor’s formal training seminar. The seminar will be conducted by Ichor personnel and will require approximately 60-90 minutes to complete. Topics will include setup and operation of the equipment, procedures for administration of the DNA vaccine, troubleshooting, and device care and storage. Each participant must successfully complete at least three mock administration procedures in order to demonstrate competency.

No more than one volunteer per day will be enrolled for the first four volunteers to receive electroporation in each dose group. For these four volunteers, an additional telephone call to assess safety and adverse events will be made on the day following vaccination. If four vaccinations by electroporation have occurred without greater than moderate adverse events, the remainder of the group can be enrolled with no fixed interval between volunteers.

All intramuscular vaccinations will be given by skilled nursing staff or physicians using sterile technique. The phlebotomy procedures will be performed by skilled professionals using sterile technique. The site of each vaccination will be given in alternating upper arms. No more than 550 mL of blood will be taken from an individual over an 8-week period. If a female subject becomes pregnant during the course of the study she will be followed up through to the end of the pregnancy (either birth of the baby or termination of the pregnancy). If the pregnancy results in birth, a pediatrician will examine the baby approximately 2-4 weeks after birth.

16. **Potential Benefits**: Participants may expect no benefit from participation in this study. Subjects will receive information about their general health and HIV status and will receive HIV counseling. The information gained from participating in this study may possibly contribute to the establishment of a vaccine strategy that may subsequently prove to reduce the transmissibility of HIV-1 infection.

17. **Risk to Benefit Ratio**: Given the experience to date with ADVAX as well as electroporation in humans and animals, and the need to develop effective vaccines to prevent HIV infection, the risk to benefit ratio is appropriate.

18. **Compensations**: Participants will receive $100 per visit to compensate for travel expenses, time lost from employment, child-care costs and other expenses they may have incurred while participating in this study. Participants will receive a check for their participation after their 2 month, 4 month and 6 month clinic visit. If a participant is unable to complete the entire study, he/she will be paid for the portion of the study that he/she completed.

Principal Investigator's Assurance:

As Principal Investigator of this study, I assure the IRB that, as required by federal regulations:

• Proposed changes in approved studies will be presented to the IRB for review and approval prior to initiation except where necessary to eliminate apparent immediate hazards to the subjects.

• The IRB, appropriate institutional officials, the Office for Protection from Research Risks (OPRR) and the FDA, if applicable, will be promptly informed of any unanticipated problems involving risks to subjects or others and research related injuries.

• The informed consent of the subject will be obtained by the investigator in the manner and format approved by the IRB prior to the initiation of the studies.

• The study will be resubmitted to the IRB for continuing review at the interval determined by the IRB to be appropriate to the risk, but not less than once a year.

|  |  |
| --- | --- |
|  | _________________________________(Signature of Principal Investigator) |

|  | March 9, 2009 |
| --- | --- |
|  | (Date) |

**REFERENCES**

***Note: Full citation of references in bold can be found in Appendix D***

1. AIDS Epidemic Update December 2001. Joint UNAIDS/WHO, December 2001.

2. Tang DA, DeVit M, Johnston SA. Genetic immunization is a simple method for eliciting an immune response. Nature 1992; 356:152-154.

3.  **Liu MA, Yasutomi Y, Davies ME, Perry HC, Freed DC, Letvin NL, et al. Vaccination of mice and nonhuman primates using HIV-gene-containing DNA. Anitbiot Chemother 1996; 48:100-104. Hanke T, McMichael AJ. Design and construction of an experimental HIV-1 vaccine for a year 2000 clinical trial in Kenya. Nat Med 2000; 6:951-955.**

4. **Wang B, Boyer J, Srikantan V, Ugen K, Gilbert L, Phan C, et al. Induction of humoral and cellular immune responses to the human immunodeficiency type 1 virus in non-human primates by in vivo DNA innoculation. Virology 1995; 211:102-112.**

5. Boyer JD, Wang B, Ugen KE, Agadjanyan M, Javadian A, Frost P, et al. In vivo protective anti-HIV immune responses in non-human primates through DNA immunization. J Med Primatol 1996; 25:242-250.

6. Lu S, Manson K, Wyand M, Robinson HL. SIV DNA vaccine trial in macaques: post-challenge necropsy in vaccine and control groups. Vaccine 1997; 15:920-923.

7. Calarota S, Bratt G, Nordlund S, Hinkula J, Leandersson AC, Sandstrom E, et al. Cellular cytotoxic response induced by DNA vaccination in HIV-1 infected patients. Lancet 1998; 351:1320-1325.

8. **MacGregor RR, Boyer JD, Ugen KE, Lacy KE, Gluckman SJ, Bagarazzi ML, et al. First human trial of a DNA-based vaccine for treatment of human immunodeficiency virus type 1 infection: safety and host response. J Infec Dis 1998; 178:92-100.**

9. Schneider R, Campbell M, Nasioulas G, Felber BK, Pavlakis GN. Inactivation of the human immunodeficiency virus type 1 inhibitory elements allows Rev-independent expression of Gag and Gag/protease and particle formation. J Virol 1997 Jul; 71(7): 4892-4903.

10. **Kotsopoulou E, Kim VN, Kingsman AJ, Kingsman SM, Mitrophanous KA. A Rev-independent human immunodeficiency virus type 1 (HIV-1)-based vector that exploits a codon-optimized HIV-1 gag-pol gene. J Virol 200 May; 74(10):4839-4852.**

11. **Haddad D, Liljeqvist S, Stahl S, Andersson I, Perlmann P, Berzins K, Ahlborg N. Comparitive study of DNA-based immunization vectors: effect of secretion signals on the antibody responses in mice. FEMS Immunol Med Microbiol 1997 Jul; 18(3): 193-202.**

12. **Li Z, Howard A, Kelley C, Delogu G, Collins F, Morris S. Immunogenicity of DNA vaccines expressing tuberculosis proteins fused to tissue plasminogen activator signal sequences. Infect Immun 1999 Sep; 67(9): 4780-4786.**

13. Weiss R, Durnberger J, Mostblock S, Scheiblhofer S, Hartl A, Breitenbach M, Strasser P, Dorner F, Livey I, Crowe B, Thalhamer J. Improvement of the immune response against plasmid DNA encoding OspC of borrelia by an ER-targeting leader sequence. Vaccine 1999 Dec(10); 18(9-10): 815-824.

14. Qiu JT, Liu B, Tian C, Pavlakis GN, Yu XF. Enhancement of primary and secondary cellular immune responses against human immunodeficiency virus type 1 gag by using DNA expression vectors that target Gag antigen to the secretory pathway. J Virol 2000 Jul; 74913): 5997-6005.

15. Wang R, Doolan DL, Le TP, Hedstrom RC, Connan KM, Charoenvit Y, et al. Induction of antigen-specific cytotoxic T lymphocytes in humans by a malaria DNA vaccine. Science 1998. 282: 476-480.

16. **Le TP, Coonan KM, Hedstrom RC, Charoenvit Y, Sedegah M, Epstein JE, et al. Safety, tolerability and humoral immune responses after intramuscular administration of a malaria DNA vaccine to healthy adult volunteers. Vaccine 2000; 18: 1893-1901.**

17. Boyer JD, Cohen AD, Vogt S, Schumann K, Nath B, Ahn L, et al. Vaccination of seronegative volunteers with a human immunodeficiency virus type 1 env/rev DNA vaccine induces antigen-specific proliferation and lymphocyte production of beta-chemokines. J Infec Dis 2000; 181: 476-483.

18. Tellez I et al. HIV-1 specific T-cell responses in seronegative volunteers immunised with an HIV-1 gag-pol DNA vaccine. 2-2-2000. 7th Conference on Retroviruses and Opportunistic Infections, San Francisco, California.

19. Donnelly, J., K. Berry, and J. B. Ulmer. . Technical and regulatory hurdles for DNA vaccines. Int J Parasitol 2003. 33:457-67

20. Somiari, S., J. Glasspool-Malone, J. J. Drabick, R. A. Gilbert, R. Heller, M. J. Jaroszeski, and R. W. Malone. . Theory and in vivo application of electroporative gene delivery. Mol Ther 2000. 2:178-87.

21. Gothelf, A., L. M. Mir, and J. Gehl. Electrochemotherapy: results of cancer treatment using enhanced delivery of bleomycin by electroporation. Cancer Treat Rev 2003. 29:371-87

22. Byrne CM, Thompson JF. Role of electrochemotherapy in the treatment of metastatic melanoma and other metastatic and primary sking tumors. Expert Rev Anticancer Ther. 2006 May; 6(5) : 671-678.

23. Babiuk, S., M. E. Baca-Estrada, M. Foldvari, M. Storms, D. Rabussay, G. Widera, and L. A. Babiuk. Electroporation improves the efficacy of DNA vaccines in large animals. Vaccine 2002 20:3399-3408.

24.  **Luxembourg, A., D. Hannaman, B. Ellefsen, G. Nakamura, and R. Bernard. Enhancement of immune responses to an HBV DNA vaccine by electroporation. Vaccine 20056. 24(21): 4490-4493.**

25. **Otten, G., M. Schaefer, B. Doe, H. Liu, I. Srivastava, J. zur Megede, D. O'Hagan, J. Donnelly, G. Widera, D. Rabussay, M. G. Lewis, S. Barnett, and J. B. Ulmer. Enhancement of DNA vaccine potency in rhesus macaques by electroporation. Vaccine 2004. 22:2489-93.**

26. **Scheerlinck, J. P., J. Karlis, T. E. Tjelle, P. J. Presidente, I. Mathiesen, and S. E. Newton. In vivo electroporation improves immune responses to DNA vaccination in sheep. Vaccine 2004. 22:1820-5.**

27. **Dupuis, M., K. Denis-Mize, C. Woo, C. Goldbeck, M. J. Selby, M. Chen, G. R. Otten, J. B. Ulmer, J. J. Donnelly, G. Ott, and D. M. McDonald. Distribution of DNA vaccines determines their immunogenicity after intramuscular injection in mice. J Immunol 2000. 165:2850-8**

28. Hirbod T, Kaul R, Reichard C, Kimani J, Ngugi E, Bwayo J, Nagelkerke N, Hasselrot K, Li B, Moses S, The Kibera HIV Study Group, MacDonald K, and Broliden K. HIV-neutralizing immunoglobulin A and HIV-specific proliferation are independently associated with reduced HIV acquisition in Kenyan sex workers. **AIDS** 2008, 22: 727.

29. Ghys P, Belec L, Diallo M, Ettiegne-Traore V, Becquart P, Maurice C, Nkengasong J, Coulibaly I, Greenberg A, Laga M, Wiktor S. Cervicovaginal anti-HIV antibodies in HIV-seronegative female sex workers in Abidjan, Cote d’Ivoire. **AIDS** 2000, 14:2603.

30. Belec L, Ghys P, Hocini H, Nkengasong J, Tranchot-Diallo J, Diallo M, Ettiegne-Traore V, Maurice C, Becquart P, Matta M, Si-Mohamed A, Chomont N, Coulibaly I, Wiktor S, Kazatchkine M. Cervicovaginal Secretory Antibodies to Human Immunodeficiency Virus Type 1 (HIV-1) that Block Viral Transcytosis through Tight Epithelial Barriers in Highly Exposed HIV-1 Seronegative African Women. **JID** 2001, 184:1412.

31. Dorrell L, Hessell AJ, Wang M, et al. Absence of specific mucosal antibody responses in HIV-exposed uninfected sex workers from the Gambia. **AIDS** 2000; 14: 1117.

32. Raux M, Finkielsztejn L, Salmon-Ceron D, Bouchez H, Excler J, Dulioust E, Grouin J, Sicard D, Blondeau C. Development and standardization of methods to evaluate the antibody response to an HIV-1 candidate vaccine in secretions and sera of seronegative vaccine recipients. **J Immunol Methods** 1999, 222: 111.

33. Mestecky J, Jackson S, Moldoveanu Z, Nesbit L, Kulhavy R, Prince S, Sabbaj S, Mulligan M, Goepfert P. Paucity of Antigen-specific IgA responses in sera and external secretions of HIV Type-1 Infected Individuals. **AIDS Research and Human Retroviruses** 2004, 20: 972.

34. Wright P, Kozlowski P, Rybczyk K, Goepfert P, Staats H, Vancott T, Trabattoni D, Sannella E, Mestecky J. Detection of Mucosal Antibodies in HIV Type-1 Infected Individuals. **AIDS Research and Human Retroviruses 2002**, 18: 1291.

35. Cole, J. and Skopek, T.R. International Commission for Protection Against Environmental Mutagens and Carcinogens. Working paper no. 3. Somatic mutant frequency, mutation rates and mutational spectra in the human population in vivo. Mutat Res, 1994. 304, 33-105.

36. Bigbee, W.L., Wyrobek, A.J., Langlois, R.G., Jensen, R.H. and Everson, R.B. The effect of chemotherapy on the in vivo frequency of glycophorin A 'null' variant erythrocytes. Mutat Res 1990. 240, 165-75.

37. Grist, S.A., McCarron, M., Kutlaca, A., Turner, D.R. and Morley, A.A. In vivo human somatic mutation: frequency and spectrum with age. Mutat Res 1992. 266, 189-96.

38 Janatipour, M., Trainor, K.J., Kutlaca, R., Bennett, G., Hay, J., Turner, D.R. and Morley, A.A. Mutations in human lymphocytes studied by an HLA selection system. Mutat Res 1998. 198, 221-6.

**APPENDIX A: SCHEDULE OF PROCEDURES**

| Visit Day (D)/Week (W) | Screen D – 42 | D  0 | **D3 Phone | Wk 1 | Wk 2 | Wk4 | Wk  8 | D 59  Phone | Wk 9 | Wk  10 | Wk  12 | Wk  16 | Wk  24 | Wk 36 | D 255 Phone^+^ | Wk 37^+^ | Wk 38^+^ | Wk  40 | Wk 44^+^ | | Wk  48 | | Wk  56 |
| --- | --- | --- | --- | --- | --- | --- | --- | --- | --- | --- | --- | --- | --- | --- | --- | --- | --- | --- | --- | --- | --- | --- | --- |
| Visit Windows  (+ Days) |  |  | 2 | 2 | 2 | 4 | 4 | 2 | 2 | 2 | 4 | 7 | 7 | 7 | 2 | 2 | 4 | 7 | 7 | 7 | | 7 | |
| Informed Consent | **X** |  |  |  |  |  |  |  |  |  |  |  |  |  |  |  |  |  |  |  | |  | |
| ADVAX Vaccine |  | **X** |  |  |  |  | **X** |  |  |  |  |  |  | **X^+^** |  |  |  |  |  |  | |  | |
| Medical History | **X** | **X** |  | **X** | **X** | **X** | **X** |  | **X** | **X** | **X** | **X** | **X** | **X** |  | **X^+^** | **X^+^** | **X^+^** | **X^+^** | **X** | | **X** | |
| General Physical Exam | **X** |  |  |  |  |  |  |  |  |  |  |  |  |  |  |  |  |  |  |  | |  | |
| Directed Physical Exam |  | **X** |  |  |  | **X** | **X** |  |  |  | **X** |  | **X** | **X** |  |  | **X^+^** | **X^+^** | **X^+^** | **X** | | **X** | |
| Pregnancy Test (women) | **X** | **X** |  | **X** | **X** | **X** | **X** |  | **X** | **X** | **X** | **X** | **X** | **X** |  | **X^+^** | **X^+^** | **X^+^** | **X^+^** | **X** | | **X** | |
| Pregnancy Avoidance Counseling | **X** | **X** |  | **X** | **X** | **X** | **X** |  | **X** | **X** | **X** | **X** | **X** | **X** |  | **X^+^** | **X^+^** | **X^+^** | **X^+^** | **X** | | **X** | |
| Risk Reduction and Safe Sex Counseling | **X** | **X** |  | **X** | **X** | **X** | **X** |  | **X** | **X** | **X** | **X** | **X** | **X** |  | **X^+^** | **X^+^** | **X^+^** | **X^+^** | **X** | | **X** | |
| HIV Testing (ref 11.1) Pre-/Post HIV test Counseling ^b^ | **X** | **X** |  |  |  | **X** | **X** |  |  |  | **X** |  | **X** | **X** |  |  |  |  |  | **X** | | **X** | |
| Adverse Events |  | **X** | **X** | **X** | **X** | **X** | **X** | **X** | **X** | **X** | **X** | **X** | **X** | **X** | **X^+^** | **X^+^** | **X^+^** | **X^+^** | **X^+^** | **X** | | **X** | |
| Serious Adverse Events |  | **X** | **X** | **X** | **X** | **X** | **X** | **X** | **X** | **X** | **X** | **X** | **X** | **X** | **X^+^** | **X^+^** | **X^+^** | **X^+^** | **X^+^** | **X** | | **X** | |
| Vital Signs (pre and 30-45 minute post vaccination) |  | **X** |  |  |  |  | **X** |  |  |  |  |  |  | **X^+^** |  |  |  |  |  |  | |  | |
| Local and Systemic Reactogenicity Assessment (pre & post vaccination) |  | **X** | **X** | **X** | **X** | **X** | **X** | **X** | **X** | **X** | **X** |  |  | **X^+^** | **X^+^** | **X^+^** | **X^+^** | **X^+^** | **+** | **+** | |  | |
| Hep Bs Ag, Hep C ABS, Syphilis | **X** |  |  |  |  |  |  |  |  |  |  |  |  |  |  |  |  |  |  |  | |  | |
| Hematology and Clinical Chemistry | **X** | **X** |  |  | **X** | **X** | **X** |  |  | **X** | **X** | **X** | **X** | **X** |  |  | **X^+^** | **X^+^** |  | **X** | | **X** | |
| Anti-DS DNA |  | **X** |  |  |  |  |  |  |  |  |  | **X** |  |  |  |  |  | **X** |  | **X** | |  | |
| Urine dipstick | **X** | **X** |  |  | **X** | **X** | **X** |  |  | **X** | **X** | **X** | **X** | **X** |  |  | **X^+^** | **X^+^** | **X^+^** | **X** | | **X** | |
| Immunology (CD4/CD8) |  | **X** |  |  |  |  |  |  |  |  |  |  | **X** |  |  |  |  |  |  |  | | **X** | |
| PBMCs for Cellular immunogenicity assays (ELISPOT&CFC) and storage |  | **X** |  | **X** | **X** | **X** | **X** |  | **X** | **X** | **X** | **X** | **X** | **X** |  | **X^+^** | **X^+^** | **X^+^** | **X^+^** | **X** | | **X** | |
| Binding/ Neutralizing Antibody Responses |  | **X** |  | **X** | **X** | **X** | **X** |  | **X** | **X** | **X** | **X** | **X** | **X** |  | **X^+^** | **X^+^** | **X^+^** | **X^+^** | **X** | | **X** | |

ET = Early Termination

^b^ Post-test counseling will be offered after HIV test results are available, at the next study visit or when available according to the site’s SOPs

** The first four subjects in each dose group who receive their vaccination by electroporation, an additional telephone call to assess safety and adverse events will be made on the day following the vaccination

**^+^**Vaccination on Week 36 and follow-up visits on Weeks 37, 38, 40 and 44 are optional, and apply only to those volunteers randomized to EP in the high dose cohort who elect to receive a third vaccination

**APPENDIX B: SKIN-FOLD MEASUREMENT INSTRUCTIONS**

Instructions for use of Accu-Measure to measure skin-fold
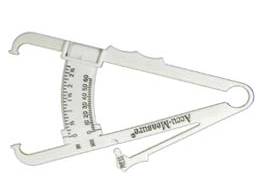
thickness

- Before starting, ensure that the sliding marker on the Accu-Measure is set to zero millimeters.
- Grasp a section of skin in the upper deltoid closest to the area of vaccination
- Holding the skin between the thumb and forefinger of one hand, and gently squeeze the Accu-Measure over that skinfold with your other hand, until you hear an audible "click"
- The sliding marker on the Accu-Measure will indicate the exact thickness of your skinfold in millimeters
- If you are not confident about the accuracy of your measurement, reset the sliding marker to zero millimeters and repeat the procedure described above.
- Once a measurement has been obtained, use the table below to select the appropriate depth setting on the Tri-Grid Applicator
- Record skin-fold measurement in the medical record
- Wipe Acc-Measure device with an alcohol swab after each use

| Range of Measured **skin fold** Thickness [mm] | TriGrid™ Application Cartridge  Depth Selection |
| --- | --- |
| < 14 mm | A |
| 14-24 mm | B |
| 24-40 mm | C |
| >40 mm | Find alternate administration site or exclude from protocol |

**APPENDIX C: ADVERSE EVENT SEVERITY ASSESSMENT TABLE**

| **PARAMETER** | **MILD** | **MODERATE** | | **SEVERE** | |
| --- | --- | --- | --- | --- | --- |
| **General Principle for Severity Assessment** | • Mild discomfort. • Minimal or no limitation of daily activity.  • No need for medical intervention. | •Moderate discomfort.  • Some limitation of daily activities (e.g. able to work part-time)  • May require minimal or no medical intervention | | •Severe discomfort. •Marked limitation of daily activities (e.g. unable to work). •Requires medical intervention. | |
| **LOCAL REACTOGENICITY:** | | |  | |  |
| **Pain and tenderness** | Minimal discomfort associated with minimal or no limitation of use of arm | Moderate discomfort with some limitation of use of arm causing some interference with daily activities. May require single dose of analgesic or NSAID. | | Severe discomfort with significant limitation of use of arm causing marked limitation of daily activities (e.g. unable to work). Requires medical intervention (e.g. repeated doses of analgesic or NSAID). | |
| **Erythema (redness) or skin discoloration** | Light red blush up to 25% of the circumference of the upper arm | Marked redness involving up to 50% of the circumference of the arm | | Brick red involving >50% of the circumference of the upper arm | |
| **Edema (swelling)** | Light edema involving up to 25% of the circumference of the upper arm | Marked edema involving up to 50% of the circumference of the arm | | Significant edema >50% of the circumference of the upper arm | |
| **Induration** | Hardening under the skin <1.5 cm in diameter | Hardening under the skin 1.5-3.0 cm in diameter | | Hardening under the skin >3.0 cm in diameter | |
| **Skin damage (vesicle, ulcer)** | Vesicles or superficial disruption of epithelium < 1 cm | Vesicles or superficial disruption of epithelium 1-2 cm | | Full thickness disruption of the epithelium (ulceration) > 2 cm | |
| **Formation of crust, scab or scar** | Crust, scab or scar = 2cm | Crust, scab or scar 2­4cm | | Crust, scab or scar > 4cm | |

| **SYSTEMIC REACTOGENICITY** | |  |  |
| --- | --- | --- | --- |
| **Headache** | Minimal headache causing no interference with daily activities | Moderate headache causing some interference with daily activities. May require single dose of analgesic or NSAID. | Significant headache causing marked limitation of daily activities (e.g. unable to work). Requires medical intervention (e.g. repeated doses of analgesic or NSAID). |
| **Rigor (chills)** | Minimal discomfort causing no interference with daily activities | Moderate discomfort causing some interference with daily activities | Significant discomfort causing limitation with daily activities (e.g. unable to work). |
| **Malaise (generalized feeling of discomfort)** | Minimal discomfort causing no interference with daily activities | Moderate discomfort causing some interference with daily activities | Significant discomfort causing limitation with daily activities (e.g. unable to work). |
| **Fatigue (tiredness)** | Minimal discomfort causing no interference with daily activities | Moderate discomfort causing some interference with daily activities | Significant discomfort causing limitation with daily activities (e.g. unable to work). |
| **Myalgia (other than vaccination site)** | Minimal muscle pain causing no interference with daily activities | Moderate muscle pain causing some interference with daily activities. May require single dose of analgesic or NSAID. | Significant muscle pain causing marked limitation of daily activities (e.g. unable to work). Requires medical intervention (e.g. repeated doses of analgesic or NSAID). |
| **Arthralgia** | Minimal joint pain causing no interference with daily activities | Moderate joint pain causing some interference with daily activities. May require single dose of analgesic or NSAID. | Significant joint pain causing marked limitation of daily activities (e.g. unable to work). Requires medical intervention (e.g. repeated doses of analgesic or NSAID). |
| **Fever** | 37.7–38.6°C (99.8– 101.5°F) | 38.7–39.3°C (101.6– 102.7°F) | >39.4°C (>102.8°F) |
| **Nausea** | Minimal nausea causing no interference with daily activities | Moderate nausea causing some interference with daily activities.. | Significant nausea causing marked limitation of daily activities (e.g. unable to work). |

| **Vomiting** | Minimal nausea causing no interference with daily activities | Moderate nausea causing some interference with daily activities. | Significant nausea causing marked limitation of daily activities (e.g. unable to work). Requires medical intervention |
| --- | --- | --- | --- |
| **Pruritus** | Localized itching at vaccination site | Itching at the injected arm (beyond vaccination site) | Generalized itching |
| **Rash** | Localized maculopapular (non­urticarial) rash at the vaccination site area causing no interference with daily activities | Diffuse maculopapular (non-urticarial) rash at the injected arm (beyond vaccination site) causing some limitation of daily activities | Systemic maculopapular (non­urticarial) rash causing marked limitation of daily activities. Requires medical intervention |
| **Allergic reaction** | Localized urticaria at vaccination site causing no interference with daily activities | Diffuse urticaria at the injected arm (beyond vaccination site) and causing some limitation of daily activities | Systemic urticaria or angioedema causing marked limitation of daily activities. Requires medical intervention |
| **HEMATOLOGY** |  |  |  |
| Hemoglobin | 10.0 g/dL – 11.0 g/dL OR  any decrease = 2.5 g/dL | 9.0 g/dL – 9.9 g/dL OR  any decrease = 3.5 g/dL | = or <8.9 g/dL  OR  any decrease = 4.5 g/dL |
| WBC—Elevated WBC—Decreased | 13,000–14,999/mm^3^ | 15,000–19,999/mm^3^ | = or >20,000/mm^3^ |
|  | 2000–2499/mm^3^ | 1500–1999/mm^3^ | = or <1499/mm^3^ |
| Absolute Neutrophil Count | 1000–1300/mm^3^ | 750–999/mm^3^ | = or <749/mm^3^ |
| Absolute Lymphocyte Count | 600–649/mm^3^ | 500–599/mm^3^ | = or <499/mm^3^ |
| Absolute CD4 Count (HIV Negative) | 300–400/mm^3^ | 200–299/mm^3^ | = or <199/mm^3^ |
| Platelets—Decreased Platelets—Elevated | 100,000–124,999/mm^3^ | 50,000–99,999/mm^3^ | = or <49,999/mm^3^ |
|  | NA | 550,000–600,000/mm^3^ | > 600,000/mm^3^ |
| **CHEMISTRY** |  |  |  |
| BILIRUBIN (total) | 1.0–1.5 x ULN | >1.5–2.5 x ULN | >2.5 x ULN |
| CREATININE | 1.1–1.3 x ULN | >1.3–1.8 x ULN | >1.8 x ULN |
| AST (SGOT) | 1.51–3.0 x ULN | >3.0–6.0 x ULN | >6.0 x ULN |
| ALT (SGPT) | 1.51–3.0 x ULN | >3.0–6.0 x ULN | >6.0 x ULN |
|  |  |  |  |

| **URINALYSIS** | | |  | |  |
| --- | --- | --- | --- | --- | --- |
| PROTEINURIA | 1+ (30 mg/dL) | 2–3+ (100-500mg/dL) | | 4+ (> 500mg/dL) | |
| Random urine 24-hour urine | 200 mg–500 mg loss/day | > 500 mg–1.0 g loss/day | | > 1.0g loss/day | |
| HEMATURIA (in the absence of vaginal bleeding) By microscopic exam only | 1+ (ca. 5–10 Ery/mL) 6-10 Ery/hpf | 2+ (ca. 10–25 Ery/µl) >10 Ery/hpf | | 3+ (ca. 50 Ery/µl) Gross, with or without clots or RBC casts | |
| GLUCOSURIA | 1+ (30mg/dl, 2.8 mmol/L) | 2+ (100mg/dL, 5.5mmol/L) | | 3+ (300mg/dL, 17 mmol/L) | |
| LEUCOCYTURIA | 1+ (ca. 10–25 Leuco/mL) | 2+ (ca. 75 Leuco/mL) | | 3+ (ca. 500/mL) | |
| **CARDIOVASCULAR** | | | | | |
| Hypotension | Transient orthostatic hypotension with heart rate increased > 20 beats/min **OR** Decreased by >10mm Hg systolic BP, No Rx required | Symptoms **OR** BP decreased by >20 mm Hg systolic, correctable with oral fluid Rx | | IV fluid required **OR** Hospitalization | |
| Hypertension | Asymptomatic, transient increase by >20 mmHg diastolic BP **OR** >150/100 if previously normal. No treatment required. | Recurrent or persistent or symptomatic increase by >20 mmHg diastolic BP **OR** >150/100 if previously normal. Treatment required. | | More intensive treatment required **OR** more than one drug required **OR** requires acute treatment | |
| Cardiac Arrhythmia | Asymptomatic with transient dysrhythmia causing no interference with daily activities. No treatment required. | Notable symptoms causing some interference with daily activities. Non-urgent treatment required | | Symptomatic and incompletely controlled by medical or invasive treatment. | |
| Pericarditis | Minimal asymptomatic effusion | More than minimal asymptomatic effusion requiring no treatment | | Symptomatic effusion, pain, EKG changes. | |
| Hemorrhage, blood loss | Asymptomatic and requiring no therapy | Mildly symptomatic | | Gross blood loss **AND/OR** 1–2 units transfused | |

| **GASTROINTESTINAL** | |  |  |
| --- | --- | --- | --- |
| Constipation | Minimally symptomatic. No medical intervention required. | Significant abdominal pain with impaction requiring prescription | Requiring disimpaction **AND/OR** Hospital treatment |
| Diarrhea | Mild or transient or intermittent episodes (2-3/day)of unformed stools resulting in minimal or no interference with daily activities | Persistent episodes (5­10/day)of unformed-to-watery stools resulting in greater than minimal interference with daily activities | Bloody diarrhea or >10/day episodes. **AND/OR** Orthostatic hypotension **AND/OR** Electrolyte imbalance requiring IV fluid/therapy |
| Oral Discomfort/Dysphagia | Mild discomfort, no difficulty swallowing | Difficulty swallowing but able to eat and drink | Unable to swallow solids |
| **NEUROLOGICAL** | | | |
| Neuropsychiatric/mood | Mild mood alteration not interfering with daily activities. No intervention required. | Depression or anxiety symptoms causing individual to seek attention and be treated with counseling and/or pharmacotherapy | Severe mood changes requiring additional medical intervention **AND/OR** Suicidal ideation/gesture |
| Paresthesia (burning, tingling, etc.) | Minimal discomfort resulting in minimal or no interference with daily activities | Notable symptoms resulting in greater than minimal changes in daily activities | Marked and persistent discomfort resulting in significant incapacity **AND/OR** Narcotic analgesia required for symptomatic improvement |
| Neuro-motor | Mild weakness resulting in minimal or no interference with daily activities | Moderate weakness resulting in greater than minimal interference with daily activities | Significant incapacity |

| Neuro-sensory | Mild impairment (decreased sensation) resulting in minimal or no interference with daily activities | | Moderate impairment resulting in greater than minimal interference with daily activities | | Significant incapacity |
| --- | --- | --- | --- | --- | --- |
| Neuro-cerebellar | Slight incoordination **OR** Dysdiadochokinesia | | Intention tremor **OR** Slurred speech **OR** Nystagmus | | Ataxia requiring assistance to walk or arm incoordination interfering with daily life activities |
| **RESPIRATORY** | | | | | |
| Cough | Transient cough resulting in minimal or no interference with daily activities | | Recurrent or persistent cough resulting in greater than minimal interference with daily activities | | Uncontrolled cough causing significant interference with daily activities |
| Bronchospasm Acute | Transient not requiring treatment. FEV1 or peak flow reduced to 70–80% | | Treatment required ­normalizes with bronchodilator. FEV1 or peak flow 50– 69% | | More intensive treatment required ­no normalization with bronchodilator. FEV1 or peak flow < 50% |
| Dyspnea | Dyspnea on exertion (such as using stairs) | | Dyspnea with normal activity (such as walking) | | Dyspnea at rest |
| **MISCELLANEOUS** | | | | | |
| Arthritis | Mild pain with no joint swelling. No interference with daily activities. | | Moderate pain with inflammation, erythema, or joint swelling. Some interference with daily activities. | | Severe pain with inflammation, erythema, or joint swelling causing significant incapacity. |
| Eye | Symptoms resulting in minimal or no interference with daily life activities | | Notable symptoms resulting in greater than minimal interference with daily life activities | | Symptoms (such as loss of vision, clinically diagnosed uveitis, or glaucoma) resulting in significant incapacity |
| Skin (general) | Localized, asymptomatic. | | Diffuse with notable symptoms and/or some interference with daily activities. | | Generalized, marked symptoms and/or significantly interference with daily activities. |
| **ADDITIONAL LABORATORY ABNORMALITIES** | | | | | |
| Fibrinogen—Elevated | 450–600 mg/dL | 601–650 mg/dL | | > 650 mg/dL | |
| Fibrinogen—Decreased | 100–200 mg/dL | < 100 mg/dL | | < 75 mg/dL | |

| Prothrombin Time (PT) | 1.1–1.24 x ULN | 1.25–1.49 x ULN | = or >1.5 x ULN |
| --- | --- | --- | --- |
| PTT | 1.1–1.66 x ULN | 1.67–2.33 x ULN | = or >2.34x ULN |
| BUN | 25–30 mg/dL | 31–40 mg/dL | = or >41 mg/dL |
| LDH | 1.5–2.5 x ULN | 2.6–3.5 x ULN | = or >3.6 x ULN |
| Hyponatremia | 130–135 meq/L | 123–129 meq/L | = or <124 meq/L |
| Hypernatremia | 146–150 meq/L | 151–157 meq/L | = or >158 meq/L |
| Hyperkalemia | 5.0–5.5 meq/L | 5.6–6.0 meq/L | = or >6.1 meq/L |
| Hypokalemia | 3.2–3.4 meq/L | 3.0–3.1 meq/L | = or <2.9 meq/L |
| PHOSPHATE Hypophosphatemia | 2.0–2.4 mg/dL | 1.5 –1.9 mg/dL | = or <1.4 mg/dL |
| Hypocalcemia | 7.8–8.4 mg/dL | 7.0–7.7 mg/dL | = or <6.9 mg/dL |
| Hypercalcemia | 10.6–11.5 mg/dL | 11.6–12.5 mg/dL | = or >12.6 mg/dL |
| Hypomagnesaemia | 1.2 –1.4 meq/L | 0.9–1.1 meq/L | = or <0.8 meq/L |
| Hypoglycaemia | 3.1–3.6 mmol/L (55–64 mg/dL) | 2.2–3.0 mmol/L (40–54 mg/dL) | = or <2.1 mmol/lL(= or <39 mg/dL) |
| Hyperglycaemia (nonfasting and no prior diabetes | 6.5–9.0 mmol/L (116–160 mg/dL) | 9.1–14.0 mmol/L (161–250 mg/dL) | = or >14.1 mmol/l (= or >251 mg/dL) |
| Triglycerides | NA | 400–750 mg/dL | = or >751 mg/dL |
| Hyperuricemia | 7.5–10.0 mg/dL | 10.1–12.0 mg/dL | = or >12.1 mg/dL |
| GGT | 1.25–2.5 x ULN | 2.6–5.0 x ULN | = or >5.1 x ULN |
| Alkaline Phosphatase | 1.25–2.5 x ULN | 2.6–5.0 x ULN | = or >5.1 x ULN |
| Amylase | 1.1–1.5 x ULN | 1.6–2.0 x ULN | = or >2.1 x ULN |
| Pancreatic amylase | 1.1–1.5 x ULN | 1.6–2.0 x ULN | = or >2.1 x ULN |
| Lipase | 1.1–1.5 x ULN | 1.6–2.0 x ULN | = or >2.1 x ULN |

**APPENDIX D: Vaccine Tolerability Assessment Form**

Please describe how much discomfort you experienced at three different points in the procedure. For each timepoint, circle the word that best describes the discomfort you felt:

**1. When the device was placed on your skin and the vaccine was injected, *before* the electrical stimulation**

0 1 2 3 4 5

*none light uncomfortable intense severe excruciating*

**2. Immediately *after* the electrical stimulation and muscle contraction**

0 1 2 3 4 5

*none light uncomfortable intense severe excruciating*

**3. Thirty minutes after the vaccination was completed**

0 1 2 3 4 5

*none light uncomfortable intense severe excruciating*

In your opinion, would this procedure be acceptable if it protected people from getting a serious disease (such as HIV / AIDS) for which we currently do not have a vaccine?

***Yes No Not Sure***

In your opinion, would this procedure be acceptable if it was able to provide significantly improved protection against a serious disease (such as influenza) for which we already have a vaccine? ***Yes No Not Sure***

**Additional Comments or feedback, if any:**

**___________________________________________________________________________________________**

**___________________________________________________________________________________________**

Initials: __________ Date: ___________________

**APPENDIX D: Volunteer Diary Card**

**Volunteer Diary Card**

**Protocol DHO-0614-0707**

Volunteer ID Number

Date and time of injection

Date of Next Visit:

Name of Study Contact:

24 HOUR EMERGENCY telephone number to report any unusual or severe symptoms that occur before the next visit:

** Please bring this card to your next clinic visit.

If you experience any symptoms following this vaccination that persist beyond day 3 (72 hours) please indicate the severity for each one noted in the column under the day you noticed the symptom. Unless an item says MEASURE, please rate severity as “-“for none, “+” for mild, “++” for moderate, or “+++” for severe.

| **Code** | **Meaning** |
| --- | --- |
| - | Symptom not present |
| + | Mild: Transient or mild discomfort, no limitation in normal daily activity |
| ++ | Moderate: Some limitation in normal daily activity |
| +++ | Severe: Unable to perform normal daily activity |

| **Possible Symptoms** | **Date** | **Date** | **Date** | **Date** | **Date** | **Date** | **Date** |
| --- | --- | --- | --- | --- | --- | --- | --- |
| Local Symptoms  Pain (pain without area being touched) at injection site |  |  |  |  |  |  |  |
| Tenderness (pain when area being touched) at injection site |  |  |  |  |  |  |  |
| Redness (erythema) at injection site  MEASURE | mm | mm | mm | mm | mm | mm | mm |
| Skin damage (vesicles, ulcers) at injection site  MEASURE | mm | mm | mm | mm | mm | mm | mm |
| Hardness or thickening (induration) at injection site MEASURE | mm | mm | mm | mm | mm | mm | mm |
| Swelling (edema) at injection site |  |  |  |  |  |  |  |
| Formation of crust, scab or scar MEASURE | mm | mm | mm | mm | mm | mm | mm |
| Systemic Symptoms  Headache |  |  |  |  |  |  |  |
| Chills |  |  |  |  |  |  |  |
| Nausea |  |  |  |  |  |  |  |
| Vomiting |  |  |  |  |  |  |  |
| Temperature MEASURE  Measure before midnight of the date stated | °F | °F | °F | °F | °F | °F | °F |
| Feeling of general discomfort, uneasiness, a general sick feeling (malaise) |  |  |  |  |  |  |  |
| Muscle aches (myalgia) |  |  |  |  |  |  |  |
| Aching joints (arthralgia) |  |  |  |  |  |  |  |

**How to Measure Thickening and Redness and skin damage**

Check for hardness or thickening (induration), redness (erythema) and skin damage (vesicles, ulcers) at injection site on Day 1, 2 and 3 after each injection. (Day 1, 2 and 3 are 24, 48, and 72 hours after study vaccination respectively).

If you see any thickening, redness, or lump at the injection site, use the measuring tape provided to measure the WIDEST part of the response on Days 1, 2, and 3.

Record the result rounded to the *nearest 10*, for example, 0, 10, 20, or 30.

**Directions for Oral Temperature**

You will be given a supply of 3M Tempa-DOT™ Disposable Thermometers to use for recording your temperature on Day 1, 2 and 3 after each vaccination.

1. Wait at least 15 minutes before taking your temperature after exposure to cold weather, smoking, eating or drinking.
2. Place thermometer under tongue as far back as possible with dots facing either up or down.
3. Press tongue down on thermometer and keep mouth closed for 60 seconds.
4. Remove thermometer. Wait 10 seconds. Some dots may disappear as device locks in for accuracy.
5. Read the last blue dot. Ignore any skipped dot. Record temperature on the diary card.
